# The genetic legacy of the 17th-century colonial capital of St. Mary’s City

**DOI:** 10.1016/j.cub.2026.04.046

**Published:** 2026-05-14

**Authors:** Éadaoin Harney, Ethan Jewett, Steven Micheletti, Roslyn Curry, Karin S. Bruwelheide, William A. Freyman, Henry Miller, Ali Akbari, Kathryn Barca, Katarzyna Bryc, Shaeloren Deering, Samantha Ancona Esselmann, Kira Kalkus, Aisling Kearns, Alexander Moran, Dominique T. Nguyen, Iñigo Olalde, Jakob Sedig, Kendra Sirak, Kim Callan, Lora Iliev, Lijun Qiu, J. Noah Workman, Matthew Mah, Gregory Soos, 23 and Me Research Team, Swapan Mallick, Nadin Rohland, Joanna L. Mountain, Douglas W. Owsley, David Reich

**Affiliations:** 1 23andMe Research Institute, 2555 Park Blvd, Palo Alto, CA 94306, USA.; 2 Department of Human Evolutionary Biology, Harvard University, Cambridge, MA 02138, USA.; 3 Department of Anthropology, National Museum of Natural History, Smithsonian Institution, Washington, DC 20560, USA.; 4 Historic St. Mary’s City, St. Mary’s City, MD 20686, USA.; 5 Department of Genetics, Harvard Medical School, Boston, MA 02115, USA.; 6 Department of Zoology and Animal Cell Biology, University of the Basque Country UPV/EHU, 01006 Vitoria-Gasteiz, Spain.; 7 Ikerbasque–Basque Foundation of Science, 48009 Bilbao, Spain.; 8 Chronicle Heritage, Phoenix, AZ 85004, USA.; 9 Howard Hughes Medical Institute, Harvard Medical School, Boston, MA 02115, USA.; 10 Broad Institute of MIT and Harvard, Cambridge, MA 02142, USA.; 11 The members of the 23andMe Research Team are Stella Aslibekyan, Adam Auton, Robert K. Bell, Katelyn Kukar Bond, Zayn Cochinwala, Sayantan Das, Kahsaia de Brito, Emily DelloRusso, Chris Eijsbouts, Sarah L. Elson, Chris German, Julie M. Granka, Barry Hicks, David A. Hinds, Reza Jabal, Aly Khan, Matthew J. Kmiecik, Alan Kwong, Yanyu Liang, Keng-Han Lin, Matthew H. McIntyre, David Poznik, Emily Rios, Shubham Saini, Anjali J. Shastri, Jingchunzi Shi, Suyash Shringarpure, Qiaojuan Jane Su, Vinh Tran, Joyce Y. Tung, Catherine H. Weldon, Peter Wilton, and Wanwan Xu; 12 Senior authors; 13 Lead contact

**Keywords:** ancient DNA, identity-by-descent, genetic pedigrees, genealogy, restoring identity, reidentification of remains, colonial America, migration, founder populations

## Abstract

Founded in 1634, St. Mary’s City was the first English settlement in the colony of Maryland. Despite existing written records and the ability of many present-day Americans to trace their ancestry to the historic city, substantial gaps remain in our knowledge of this early founder population. To address these gaps, we analyzed the genomes of 49 individuals from 17th-century St. Mary’s City to trace their genetic ancestry, examine their enduring legacy, and demonstrate the efficacy of using an identity-by-descent (IBD) approach to link historical individuals to the present. In our analysis, we identified over 1.3 million genetic relatives of the St. Mary’s individuals among research participants in the 23andMe Research Institute’s database. We found high rates of genetic sharing with participants from western England and Wales, suggesting a likely place of origin for many of the colonial city’s earliest inhabitants. Additionally, we observed strong genetic connections with participants from Kentucky, mirroring a recorded post-Revolutionary War migration of Maryland Catholics to that region. By further integrating genealogical information from present-day research participants who share the closest genetic connections to the St. Mary’s individuals, we propose possible identities for three sequenced historical St. Mary’s City residents, including Thomas Greene, the second governor of the colony of Maryland. This unique case study highlights the power of genetics to restore lost identities and reconstruct historical relationships by tracing geographic signals of ancestry.

## eTOC Blurb

Harney et al. analyze 49 genomes from 17th-century St. Mary’s City, Maryland. Using an IBD-based approach they identify connections to over 1.3 million living relatives, enabling them to study the founders’ ancestral origins, trace descendant migrations across the US, and propose a possible identity for Maryland’s second governor.

## Introduction

Early colonial founder populations have had a lasting impact on the genetic landscape of North America, contributing disproportionately to the biogeographic ancestry of present-day people in the United States and elsewhere across the continent ^1,2^. While historical documents and genetic studies of present-day populations offer partial insights into these early groups, ancient DNA (aDNA) now enables direct investigation of the genomes of historical individuals who made up these founding populations, enabling us to address questions about their biogeographic origins, relationships, and the genetic legacy they left in present-day populations ^3^.

Identical-by-descent (IBD) segments, which are regions of DNA shared between two individuals because they were inherited from a common ancestor, serve as a key tool for studying historical populations ^4,5^. Long IBD segments are evidence of recent genealogical relationships, while shorter segments signal distant shared ancestry.

**Figure 1.**
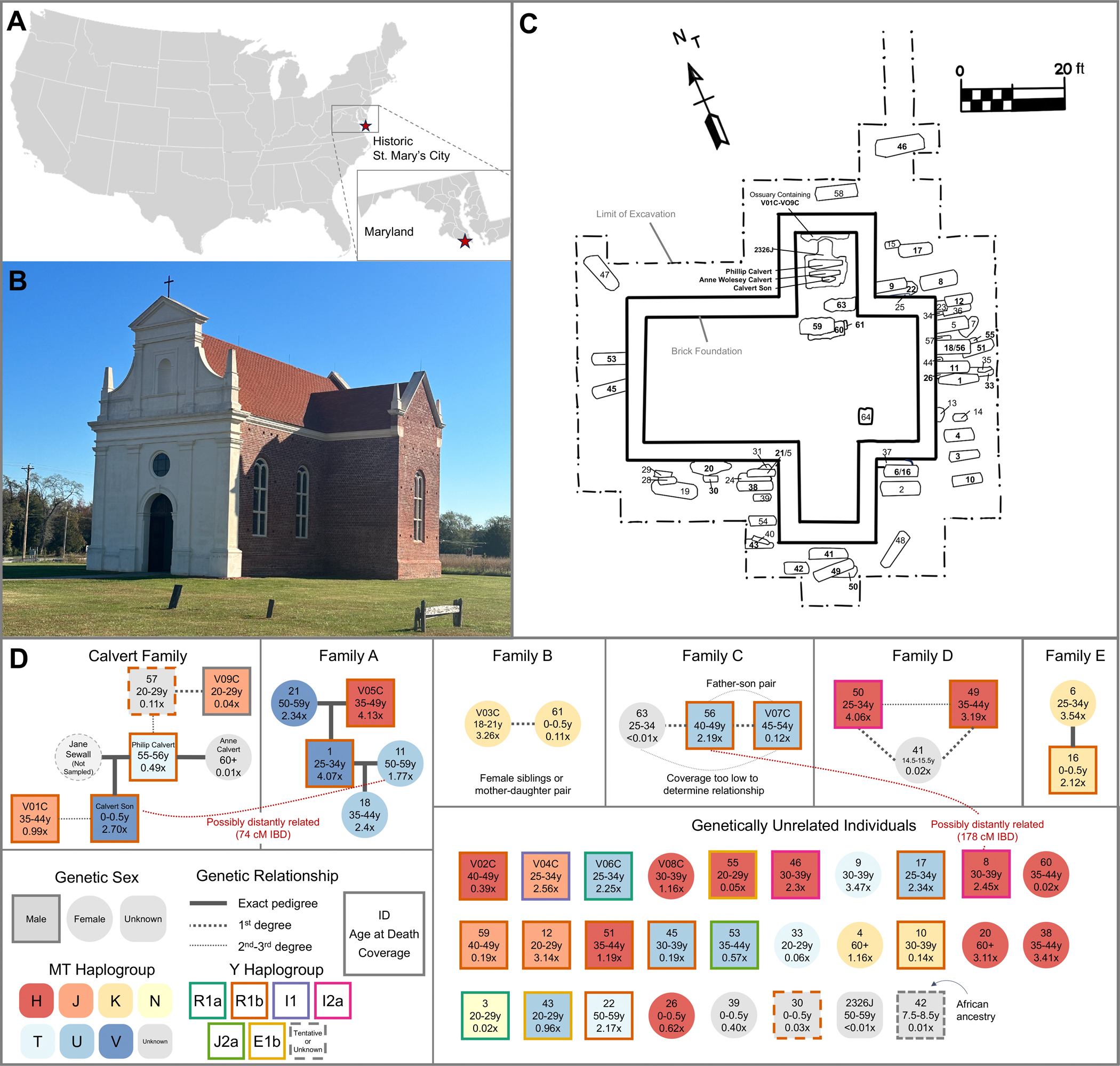
Location, genetic kinship, and haplogroups of St. Mary’s individuals. (A) Map showing the location of Historic St. Mary’s City in the United States, with a panel showing its location within Maryland. (B) Photo of the reconstructed Brick Chapel at Historic St. Mary’s City. (C) Site map showing the location of excavated burials within and near the Brick Chapel. Burials sampled for DNA are indicated in bold. (D) Family groupings were identified among the St. Mary’s individuals through genetic connections, except in the case of Anne Wolseley Calvert, who is known from historical records to have been the first wife of Philip Calvert and whose biological profile matches the remains buried at his side. Individuals are labeled according to burial ID and average chromosomal coverage. Circular and square markers are used to represent genetically female and male individuals, respectively. The fill color of each marker corresponds to the MT haplogroup, and for genetically male individuals, the outline color corresponds to the Y-chromosome haplogroup. Genetic relationships shared between individuals are indicated by connector lines. A solid dark gray line indicates the exact pedigree, with first and second-to-third-degree relatives connected by a thick and thin dotted gray line, respectively. More distant relationships detected via IBD sharing that span family groups are connected by a dotted red line. (See also Data S1, S2, and Data S7A–B)

Here we extend the aDNA IBD framework introduced in Harney et al. ^3^ to study the 17^th^-century inhabitants of St. Mary’s City, the founding English settlement in the colony of Maryland (Figure 1 A–C). Founded in 1634, the settlement drew English and some Irish immigrants of diverse religious backgrounds ^6^. Despite existing primary source documents and the ability of present-day Americans to trace their ancestry to St. Mary’s City, substantial gaps remain in our knowledge of this early founder population, including aspects of identity of those buried in the Chapel Field cemetery (Data S1).

To date, only three individuals interred in the Chapel Field cemetery have been identified with confidence: Philip Calvert, his first wife Anne Wolseley Calvert, and Philip’s infant son with his second wife, Jane Sewell ^7,8^. Even for the prominent Calvert family, the burial locations of many of its members are unrecorded, including Philip’s half-brother, Leonard Calvert (1610–1647), who served as the first governor of Maryland ^9^. Even less is recorded for the remainder of the historic city’s ancestral families. Thus, despite its relatively rich historical record, St. Mary’s City presents unresolved questions ideally suited to genetic investigation, particularly regarding origins, kinship, and identity.

The ability of aDNA to provide insights into the biogeographic origins of historical individuals and the relationships they share with one another is well established ^10^. However, whether genetic data can be used to restore the identities of individuals who have no surviving names in the documentary record is less well explored. Previous aDNA studies that made genealogical connections between historical and living people relied on strong prior hypotheses based on archaeological or archival evidence and often could only be applied to questions of very recent descent ^11,12^. By contrast, the IBD-based approach we introduce in this study does not require a candidate identity nor is it limited to the closest genealogical connections. Instead, by searching for IBD between historical individuals and millions of living research participants, we can identify multiple present-day relatives who are genetically connected (likely by 9 or more degrees) to the same historical individuals. Integrating information gathered from their genealogical pedigrees, we use a triangulation approach to converge on possible identities for previously unnamed historical individuals without any initial hypothesis–a capability that significantly expands what genetic analyses can reveal about the past.

We generated genome-wide data from 49 individuals excavated from the Chapel Field cemetery at St. Mary’s City and examined IBD connections to 11,524,442 genotyped research participants from 23andMe Research Institute, including more than 14,000 with ancestral ties to the settlement. These comparisons enable us to characterize the biogeographic origins of the St. Mary’s individuals, reconstruct family relationships within the cemetery, document their genetic legacy among present-day Americans, and present a case study that proposes possible identities for individuals whose names were otherwise unknown.

These results show how documented historical migrations are reflected in the genomes of present-day Americans and demonstrate that IBD analysis can add to our understanding of even well-studied historical populations. Our analysis of St. Mary’s City thus serves as a test case for applying this approach to other, less well-understood sites, offering a framework for reconstructing population history, genealogy, and identity in the past.

## Results

### Genetic Relationships Observed Among the St. Mary’s Individuals

From a total of 65 individuals recovered from single interments and a commingled ossuary associated with the Brick Chapel at St. Mary’s (c.1636–1730), we selected 50 individuals for genetic analysis. We generated genome-wide data for 49 of these individuals at 1.24 million variable DNA positions, with a median per-individual average chromosomal coverage of 1.16x (range: <0.01x–4.13x) (Data S7A). We identified six genetic families by comparing pairwise mismatch rates in pseudohaploid genotypes between individuals (Figure 1D). Five genetic families were identified exclusively using genetic connections, while the sixth was identified via a mixture of historical records, osteological analysis, and genetic connections. We use the term genetic family to refer to groups of individuals who share a close genetic relationship. Similarly, we use specific relationship terms (e.g., mother, father, son, daughter) in the biological sense, acknowledging that these genetic relationships may not directly align with the kin-based relationships or gender-identities recognized by the St. Mary’s individuals.

Family A is the largest newly identified genetic family, consisting of a genetically female adult, her parents, and her paternal grandparents. Families B-E are each composed of two or three individuals who share first-degree relationships, but the nature of most of these relationships is less understood. For instance, based on the available sequencing coverage we could not determine whether Family B consists of a mother-daughter pair or sisters.

The Calvert family includes three individuals of known identity: Philip Calvert, his first wife Anne Wolseley Calvert, and Philip’s infant son, referred to as “the Calvert Son.” We identified three other individuals with genetic ties to Philip Calvert or the Calvert Son. V01C, represented only by a cranial vault interred within the ossuary, shares a second-to-third-degree relationship with the Calvert Son, but we detected no relationship with Philip Calvert, suggesting that the two individuals are connected via the infant’s mother, Jane Sewell. Burial 57, an individual whose burial contained only lower limb bones (a result of the disturbance of the grave during construction of the Chapel foundation), is a second- to third-degree relative of Philip Calvert and shares a first-degree relationship with V09C, another individual whose cranial vault was interred in the ossuary. While no genetic connection was detected between Philip Calvert and V09C, we cannot exclude the possibility that they were related, given the poor preservation and elevated rate of contamination detected in V09C. In fact, this elevated contamination rate makes it impossible to rule out that V09C and Burial 57 are the same individual. We discuss their possible relationship to Philip Calvert further in Data S2.

**Figure 2.**
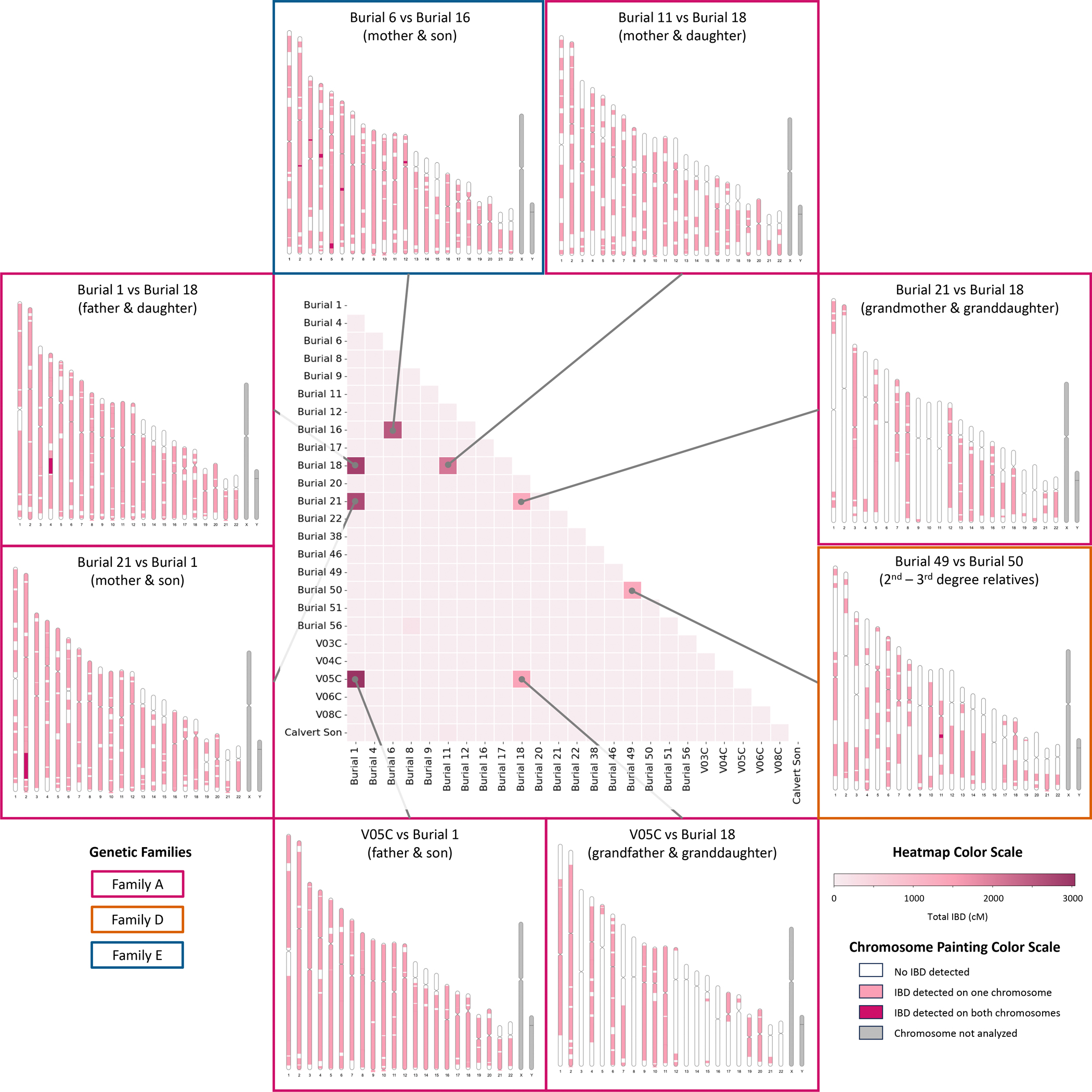
IBD sharing between the St. Mary’s individuals. A heatmap showing total IBD shared between all pairs of St. Mary’s individuals with at least 1x average chromosomal coverage. Karyotype plots showing the location of IBD segments detected between pairs who share the greatest amount of IBD surround the heatmap. Light pink indicates genomic regions where a single IBD segment was detected, and darker pink marks regions with two overlapping segments. Chromosomes X and Y are shown in grey to indicate that they were not included in the IBD analyses. We note that consecutive short IBD segments within a chromosome likely result from phase switch errors, and that the true IBD segments probably span these closely located regions. (See also Data S7B)

For the 25 historical individuals with sufficient DNA preservation to enable IBD analysis (i.e., at least 1x average chromosomal coverage), the IBD sharing between the individuals supports the inferred relationships (Figure 2, Data S7B). Using IBD sharing patterns, we clarified the relationship between Burials 6 and 16 of Family E, who we conclude were mother and son. Our IBD analysis also suggested that some of the individuals had more distant relationships to one another. For instance, Burial 11 and Burial 18–the mother-daughter pair from Family A–share up to 100 centimorgans (cM) of IBD with the Calvert Son, suggesting that they were distant relatives. Similarly, we detected 178 cM of IBD between Burial 56 of Family C and Burial 8.

### The Genetic Ancestry of the St. Mary’s Individuals

We found that the St. Mary’s individuals in our study have entirely European-related ancestry, except for Burial 42, who has primarily African-related ancestry, with a smaller proportion (25–30%) of European-related ancestry (Figure S1). This young boy, who was wrapped in a shroud and buried in a gable-lidded coffin, has poor skeletal and DNA preservation and therefore could not be included in subsequent analyses.

To explore the biogeographic ancestry of the 25 St. Mary’s individuals of European descent whose genetic data could be confidently imputed (i.e., those with >1x average chromosomal coverage), we searched for IBD connections to 11,524,442 23andMe research participants using the approach established in Harney et al. ^3^. We found that 11.85% of all research participants share IBD with one or more of the St. Mary’s individuals, with up to 150 cM shared with a single St. Mary’s individual: Burial 1, an American-born, young adult male and member of Family A (Table 1, Figure S2, Data S7C–D). The highest rates of IBD sharing occur among participants from regions where British and Irish ancestries are well represented (Figures S3–4, Data S7E), including northwestern Europe, the US, South Africa, and Australia, reflecting distant shared ancestry.

The 25 St. Mary’s individuals included in IBD analyses exhibit a high degree of variability in coverage (1.16–4.13x). Individuals with less than 2x average chromosomal coverage tended to share the fewest IBD connections––an expected consequence of the higher minimum IBD segment length threshold (9 cM) applied to individuals with <2x coverage (versus 6 cM for individuals with 2–5x coverage). In Data S3 we explore how variability in coverage impacts IBD sharing rates for historical individuals that were subject to the same segment length filtering thresholds by downsampling higher coverage individuals to 2x coverage. We found that although the IBD sharing rate increases with higher coverage, coverage is not the sole driver of differences in IBD sharing patterns observed across historic-era individuals. Instead, differences also appear to reflect real differences in their genetic legacy (Figure S5).

### Genetic connections in Great Britain and Ireland

Within Europe, the St. Mary’s individuals exhibit a high rate of sharing with participants from Great Britain and Ireland (26.2%). Using randomization testing, we confirmed that we would be unlikely to observe this frequency of sharing within an identically sized sample of participants randomly selected from across Europe with at least 99% European ancestry (among whom the overall rate of sharing is 13.92%). This pattern therefore likely reflects a historically significant connection to the region (p-value <0.001) (Data S7F).

**Figure 3.**
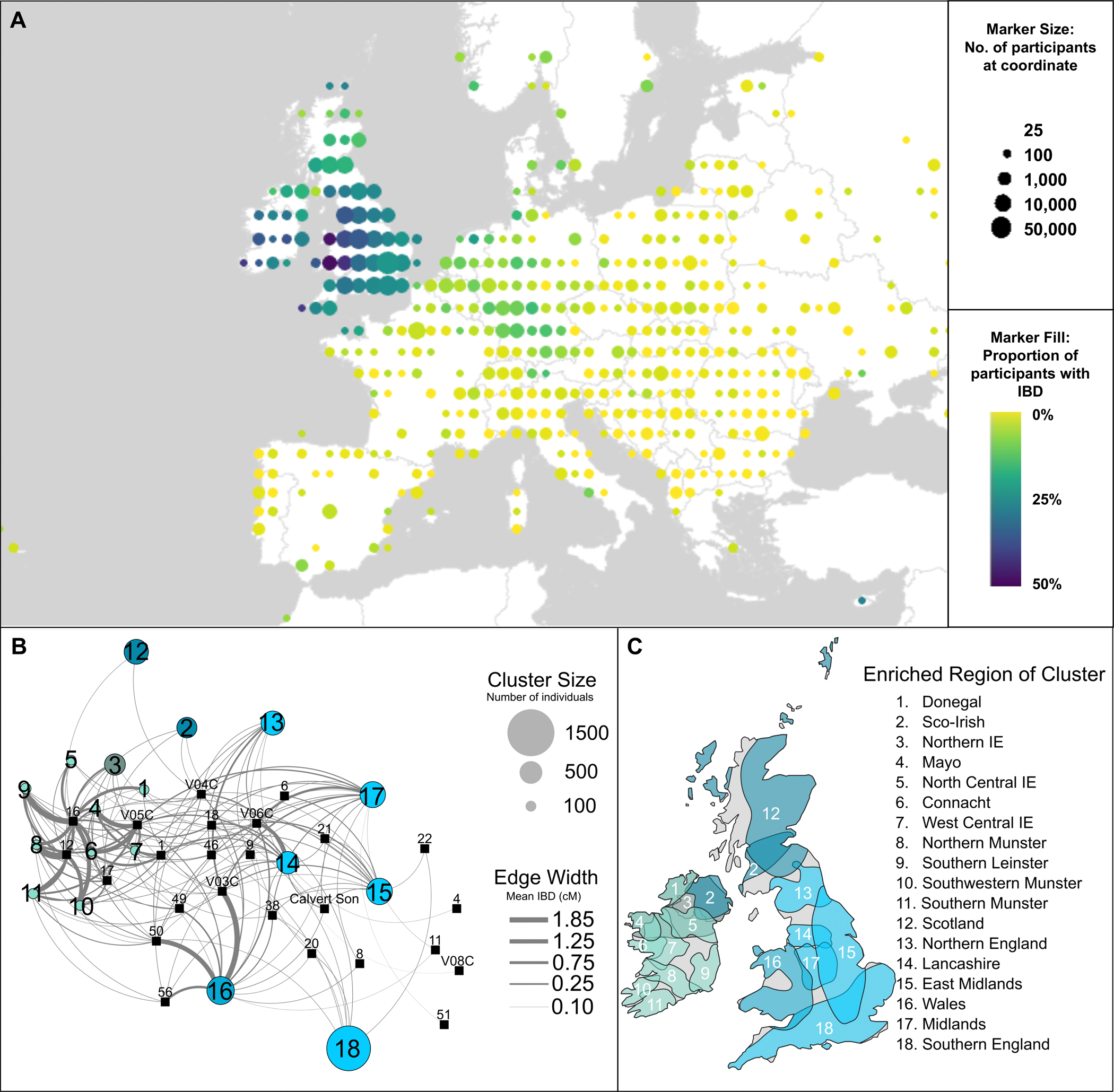
Genetic connections to the St. Mary’s individuals among research participants from Great Britain and Ireland. (A) A map of Europe in which marker color indicates the proportion of research participants with at least 99% European ancestry at each geographic coordinate who share IBD with one or more of the St. Mary’s individuals. The size of each marker represents the number of participants at the given geographic coordinate (rounded to the nearest integer). To protect participant privacy, we randomly downsampled to include only 80% of participants and only showed results for coordinates with at least 25 associated participants. (B) IBD network demonstrating St. Mary’s individuals’ connections to genetic groups in Great Britain and Ireland (N = 7,872). Each circle represents a genetic group that was identified using stochastic block modeling ^24^ on IBD connections between individuals that have all 4 of their grandparents born in either Great Britain or Ireland. The size of each genetic group is scaled by the total number of individuals assigned to it (ranging from 88 – 1457). Genetic groups are arranged by the average pairwise IBD sharing between clusters (edges not shown) using a Force Atlas graph layout. St. Mary’s individuals, displayed as squares, are projected over the graph network and arranged based on the average IBD that is shared with each cluster (shown as lines; edges smaller than 0.10 cM are not shown). (C) The geographic distribution of genetic clusters in Great Britain and Ireland. Ranges represent the limits of kernel density estimates based on coordinates of self-reported grandparent locations from individuals in each cluster. (See also Data S7G–H, Figure S4–S5)

When all 25 St. Mary’s individuals were considered together, we observed the highest rates of sharing with participants from Wales and western England, particularly the Western Midlands (Figure 3A, Figure S5, Data S7G). Comparing the St. Mary’s individuals to genetic clusters composed of participants from Great Britain and Ireland corresponding with geography (Figure 3B), we found that multiple St. Mary’s individuals share IBD with cluster 16, a group composed of participants with ties to Wales and surrounding areas in England. Applying the same randomization framework, we found that the frequency of IBD sharing with participants who make up this cluster (51.84%) was far greater than expected given the overall sharing rates observed among participants with at least 99% Northern European ancestry randomly sampled from across Great Britain and Ireland (26.77%; p < 0.001). Collectively, these results suggest that the sequenced St. Mary’s individuals had ancestral ties to Wales and western England.

The strongest connections to the Welsh genetic cluster (cluster 16) were observed among Burials 49, 50, 56, and V03C (Figure 3B–C, Figure S5, Data S7H). V06C also shares substantial IBD with participants from cluster 16, but with even stronger connections to clusters 14 and 17, composed of participants from the Lancashire and Midlands regions of England, respectively. In contrast, five individuals (Burials 1, 12, 16, 17, and V05C) show greater sharing with genetic clusters in Ireland than Great Britain, suggesting that this is the source of their ancestry.

### Genetic connections in the United States

**Figure 4.**
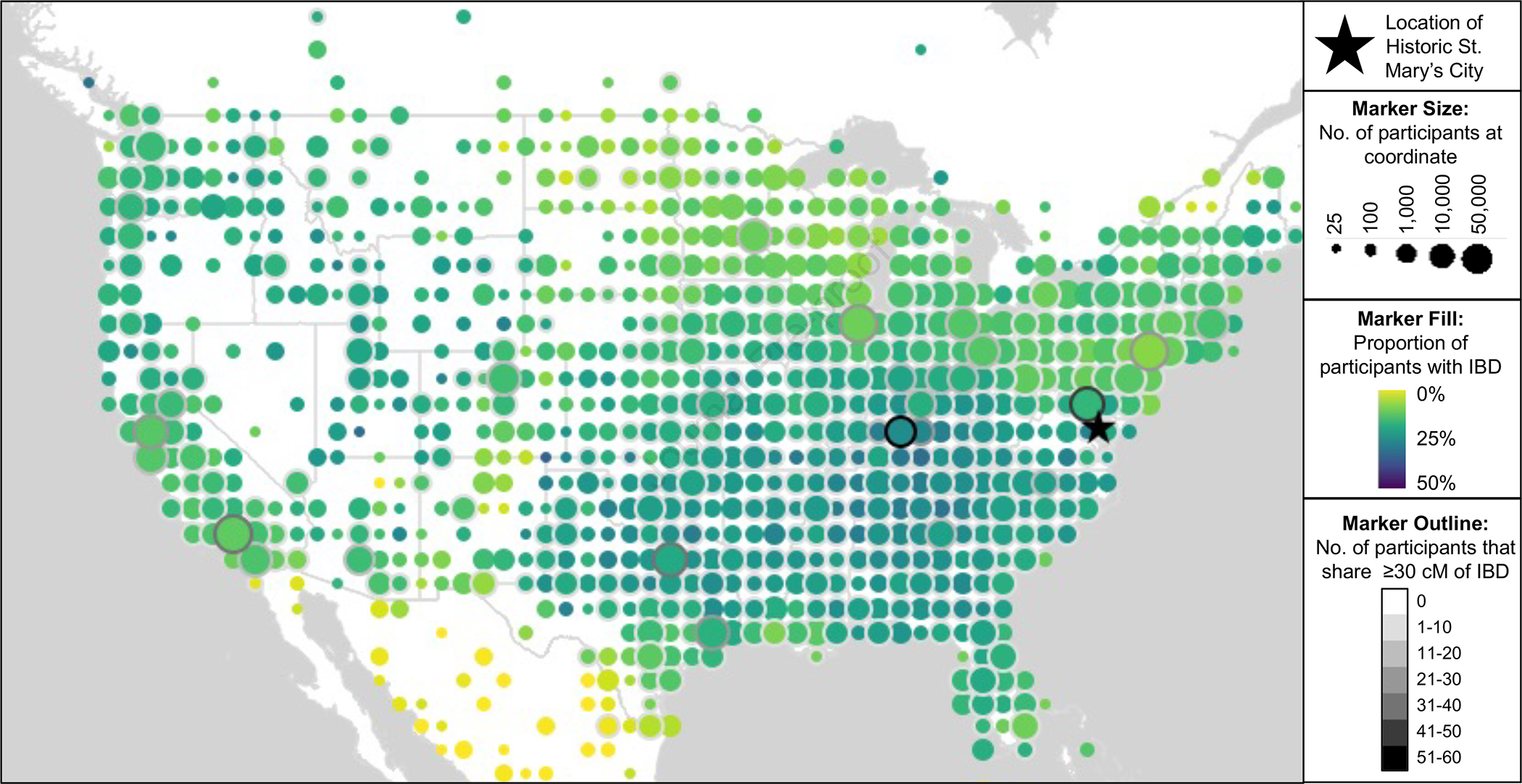
Genetic connections to the St. Mary’s individuals among research participants in the US. Marker color indicates the proportion of research participants in the US at each geographic coordinate who share IBD with one or more of the St. Mary’s individuals. The size of each marker represents the number of participants at the given geographic coordinate (rounded to the nearest integer). To protect participant privacy, we randomly downsampled to include only 80% of participants and only show results for coordinates with at least 25 associated participants. Marker outline colors indicate the number of participants at each location who share at least 30 cM of IBD with one or more St. Mary’s individuals. The star indicates the location of St. Mary’s City, Maryland. (See also Data S7E, Figure S5.)

The St. Mary’s individuals share IBD with 15.48% of participants in the US (Table 1), particularly those in Southern states (Figure 4, Figure S5, Data S7F). We confirmed via randomization testing that there is a significantly higher rate of sharing among participants with 99% European ancestry in Southern states than in other US regions (20.1% vs 17.22%; p-value <0.001), suggesting that this finding may reflect settlement patterns of individuals of European ancestry in the early US (Data S7G). This signal is broadly consistent across all St. Mary’s individuals, although the intensity of this pattern varies among individuals. Differences in coverage only partially explain this, with the lowest coverage individuals exhibiting the lowest rates of sharing (Figure S5).

### Close relatives

We next studied participants with the strongest genetic connections to the St. Mary’s individuals by focusing on pairs of historical individuals and research participants who shared at least 30 cM of IBD (referred to as “close relatives”). Given that most of the St. Mary’s individuals died during the 17^th^ century, the most likely predicted relationship between pairs who share 30 cM of BID on up to 3 segments is less than 17 degrees. Due to the random way in which DNA is inherited, there is a large amount of variation associated with this prediction, meaning that it cannot be assumed that all participants who share 30 cM of IBD with the St. Mary’s individuals are direct descendants. Instead, they could be collateral relatives whose direct ancestors were closely related to the St. Mary’s individuals, with a shared common ancestor within a few generations. In fact, as we expect that the St. Mary’s individuals have significantly more collateral relatives living today than direct descendants, our approach for identifying genetic relatives may be subject to the “winner’s curse,” whereby we detect an overabundance of distant relatives who share unusually large amounts of DNA from their distant historical relatives compared to the number of true direct descendants. In Data S4 we discuss IBD-based relationship inference in more detail.

We detected 81 close relatives with ties to Maryland, suggesting that descendants and close collateral relatives of the St. Mary’s individuals remain in the region (Figure 4, Data S7I). However, the largest number of close relatives report ties to Kentucky (n=211), particularly in areas surrounding Louisville, plausibly reflecting a documented migration of families from St. Mary’s City to Kentucky between 1780–1820 ^13–16^. This signal is particularly evident among connections to Burial 56 (part of Family C). Applying the randomization testing framework to consider the distribution of close relatives, for both Maryland and Kentucky, we would be unlikely to identify as many close relatives among an identically sized, random sample of participants selected from across the US (where we observe a total of ~2,030 close relatives), after restricting our analysis to participants with at least 99% European ancestry (Data S7F), providing further support for the historical significance of this IBD sharing pattern.

### Ancestral Ties to 17^th^-century Historic St. Mary’s City

Many people in the US who can trace their ancestry to British colonists report ancestral ties to people who lived in, or were otherwise associated with, 17th century St. Mary’s City. To determine whether people with known ties to the historic city share IBD with the St. Mary’s individuals at an elevated rate, we asked research participants to answer the question, “Can you trace your relatives back to 17^th^-century St. Mary’s City, Maryland’s first colonial settlement?” (possible answers: “Yes”, “No” and “I’m not sure”). Of the 620,260 research participants in the US cohort who responded to this question, 14,814 participants answered “Yes” (Table 1).

A significantly higher proportion of research participants in the US cohort who answered “Yes” to this question share IBD with the St. Mary’s individuals than those who answered “No” (18.54% vs 14.85%) (chi-square test, p=1.62×10^−34^; Data S7J). To ensure that this difference was not solely caused by differences in ancestry among respondents of this question, we restricted our analysis to only participants in the US cohort with at least 99% European ancestry, and we again found that “Yes” respondents share IBD with the St. Mary’s individuals at a significantly (p=1.71×10^−15^) higher rate than “No” respondents (18.95% vs 16.12%, Data S7J). These results suggest that the sharing we detect among research participants reflects recent shared ancestry in many cases. A similar pattern appears when considering mean IBD sharing between cohorts (2.33 vs 1.77 cM, Data S7J). This is expected, as most segments lie near our minimum length threshold, causing patterns in mean sharing to closely track overall sharing rates.

### Restoring identity

Next we explored whether participants’ genealogical connections to 17^th^-century St. Mary’s City could be used to re-identify any of the sequenced St. Mary’s individuals (Data S5). We collected self-reported genealogical pedigree information from consenting research participants with some of the strongest genetic connections to the St. Mary’s individuals. This work was conducted under a custom research protocol that permitted the analysis of individual-level ancestral connections.

**Figure 5.**
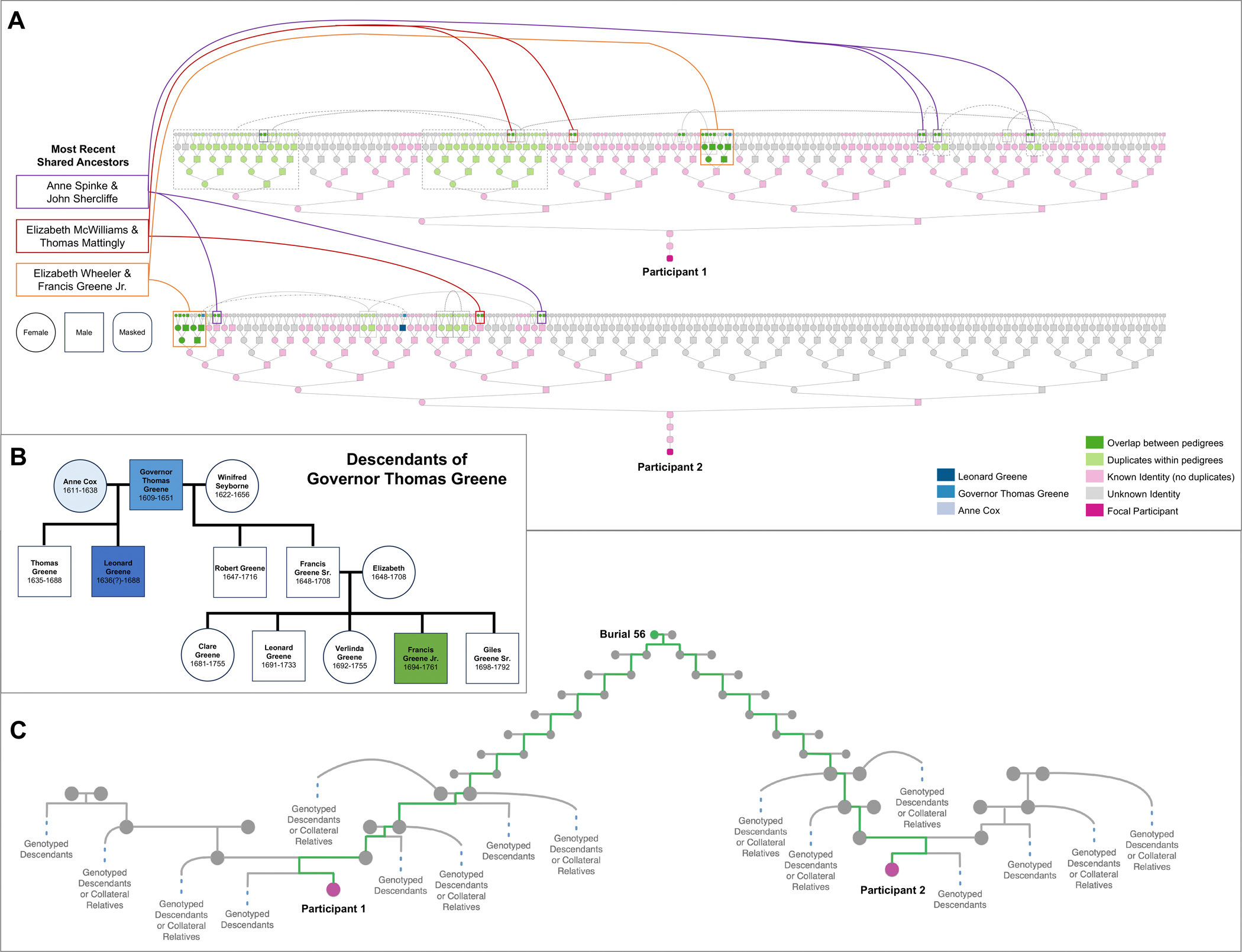
Genealogical and genetic pedigrees connecting focal participants to Burial 56. (A) The reconstructed genealogical pedigree of each focal participant who shares over 30 cM of IBD with burial 56 is shown. The pedigrees focus on ancestors of the focal participants who were born prior to 1850 on lineages with reported connections to St. Mary’s City (Data SK-L). Ancestors that appear in both pedigrees are shown in dark green (and solid lines lead to the names of these ancestors). Ancestors that appear multiple times within the same pedigree are shown in light green (and duplicate portions of the pedigrees are outlined and connected via a dotted line). Ancestors for whom an identity is known are shown in light pink. The focal participant is shown in dark pink. Individuals whose identity is unknown are shown in grey. (B) Genealogical tree showing the descendants of Governor Thomas Greene and his wives Anne Cox and Winifred Seyborne. Only the children of Francis Greene Sr. are shown, in order to demonstrate the relationship between Leonard Greene and Francis Greene Jr. (C) Genetically inferred pedigree for the two focal participants, who are shown in purple. Branches indicate the locations of sets of one or more genotyped descendants or collateral relatives. The genetically inferred position of Burial 56 is shown in green and the lineages leading from each survey participant to this node are shown in green. (See also Data S4–S6 and Data S7K–L)

We centered our analysis on Burial 56, searching for overlaps in the genealogies of two participants (1 and 2) who shared large amounts of IBD with this individual (48 and 33 cM, respectively). We hypothesized that males appearing in both trees could be candidates for Burial 56. We identified three ancestral couples that appeared in both trees (Figure 5A–B): Francis Greene Jr. (1694–<1761) and Elizabeth Wheeler (1693–1758); Thomas Mattingly (1623–1664) and Elizabeth McWilliams (1626–1714); and John Shercliffe Sr. (1618–1663) and Anne Spinke (1618–1678). All three couples share a 9th–11th degree direct-descent relationship with both participants, consistent with the maximum likelihood estimates from our genetic pedigree reconstruction (Figure 5C). While random inheritance patterns make predicting exact degrees of relatedness difficult, we prioritized these couples and their close relatives as the most likely candidates. To determine which of these candidates (if any) could be Burial 56, we compared what is known about them from the historical record with the biological profile of Burial 56, established by osteological and archaeological assessments.

Burial 56 was situated against the eastern edge of the Chapel’s foundation in a heavily used area of the cemetery. It was an unusual secondary burial, meaning the skeleton had been previously disturbed and redeposited in a wooden box. The burial was commingled, containing remains from more than one individual ^7^. Alongside the primary individual, the box contained leg bones believed to belong to Burial 57, a nearby partial grave that was disturbed by the Chapel’s construction and the later excavation of Burial 56. The elements representing Burial 56 were carefully sorted based on similarities in age, sex, preservation, and stable isotope analysis, revealing that Burial 56 was a male of European ancestry, aged approximately 40–49 years, with a dietary signal that indicated he was either born in Maryland, or immigrated there as a young child. Based on the archaeological context, he was likely buried between 1667 and 1704 (following the Chapel’s construction); however, because this was a secondary burial, his actual death date may have been earlier. We therefore considered candidates who died between 1650 and 1704.

Based on this profile, we ruled out John Shercliffe Sr. and Thomas Mattingly as candidates, as both men were born in England and immigrated as adults. While Francis Greene Jr. was born in Maryland, he was ruled out because he lived too recently–while his exact date of death is unknown, his youngest child was born in 1745, indicating that he survived at least forty years beyond the latest date associated with the cemetery’s use. After eliminating these primary candidates, we went on to investigate other members of their family trees based on information available in the FamilySearch database.

The ancestors of John Shercliffe Sr. and Thomas Mattingly (and their wives) could all be ruled out, as they were born in England. Therefore, we focused our analysis on the lineage of Francis Greene Jr.. His father, Francis Greene Sr. (1648–1708), died outside our target age and date ranges. However, Francis Greene Sr.’s half-brother, Leonard Greene (c.1636–1688), fits this profile ^17^. While his reported age at death of 50–52 is slightly older than the osteological estimate (40–49 years), it is within an acceptable margin of error for skeletal aging.

Providing further support for this identification, Leonard’s father, Governor Thomas Greene (1609–1651), fits the profile of V07C. V07C was identified as the father of Burial 56 through a combination of genetic and isotopic evidence. Genetics support a father/son relationship, while isotopes indicate that V07C was born in England and emigrated to Maryland while Burial 56 was born and raised in Maryland. Due to the locations of their births, it is far more likely that V07C is Burial 56’s father than his son. The remains of V07C were redeposited in an ossuary when the chapel was built in 1667, providing an indicator of the latest year he could have died and his age of death was estimated to be in the range of 40–54 years. Similarly, Burial 56 shares a first degree relationship with Burial 63, who fits the profile for Leonard’s mother, Anne Cox (1611–1638). We provide more details about the other candidates that we considered in Data S6. However we believe that these three individuals (Leonard Greene, Anne Cox, and Governor Thomas Greene) best fit the biological profiles of Burials 56, 63 and V07C, respectively.

Interestingly, Leonard Green is also a direct ancestor of participant 2, thereby making Governor Thomas Greene the direct ancestor of this participant at least two times over. This highlights the complex loops that are common throughout genealogical pedigrees involving the St. Mary’s founding population.

While this analysis has enabled us to propose possible identities for three St. Mary’s individuals, we caution that there are substantial gaps in the genealogical pedigrees we reconstructed based on the self-reported family histories of the two focal participants, leaving open the possibility that there are additional, unidentified, overlaps in their trees. Additionally, it is possible that Burial 56 may be a more distant collateral relative of the three focal couples we considered.

## Discussion

Although St. Mary’s City is a relatively well-documented colonial settlement, surviving records provide little information about the vast majority of its residents ^6^. This study therefore highlights the unique power of IBD-based analyses to deepen our understanding of one of the earliest British colonial communities in North America and to trace the genetic legacy of its founders.

Among the 49 St. Mary’s individuals studied all but one had exclusively European ancestry. The exception, a young boy with majority African ancestry interred among the European-ancestry individuals, following similar burial customs, is a significant finding that warrants additional consideration. His discovery is an important reminder of the diversity present in the Chapel Field cemetery.

For the European-ancestry individuals, we identified five genetic families, at least one of which spans three generations. Further, IBD sharing indicates that many of the St. Mary’s individuals included in our study likely shared more distant genetic relationships, involving four or more degrees of separation. Although Philip Calvert, his first wife Anne Wolseley Calvert, and his infant son were tentatively identified by previous archaeological, osteological, and historical research, little was known about others who were buried in the Chapel Field cemetery. Here, we identify three additional members of the Calvert extended family, including whose crania were found in an ossuary pit dating to 1667. The next largest genetic family (Family A) may also have been distantly related to the Calvert Family.

Historical records document that The *Ark* and The *Dove*, the ships carrying the first St. Mary’s colonists, set sail for the Americas from the Isle of Wight off the south coast of England ^18^. However, these records provide little information about the exact ancestral origins of the passengers or about the many subsequent vessels that carried immigrants to the colony. We show that many of the St. Mary’s individuals shared genetic connections to participants from western England and Wales, suggesting that they were common places of origin for the city’s early settlers. Although we cannot exclude the possibility that this pattern is influenced by broader demographic changes that occurred within Great Britain over the last few hundred years, fine-scale regional structure within Great Britain has been shown to persist into the present-day, despite modern mobility, supporting the use of contemporary regional patterns as a proxy for historical ones ^19^.

We also identified a number of St. Mary’s individuals who exhibited connections to other regions in Great Britain and Ireland, indicating that the St. Mary’s founding population had diverse origins from across the two islands. Notably, two individuals who exhibited strong genetic connections to Ireland (Burials 12 and 17) were young adult males who died between 1634 and 1667. Isotopic evidence indicates that both were relatively recent immigrants based on previously established patterns of colonial diet and migration ^20,21^. Neither was buried in a coffin and only one with a shroud. Both showed skeletal signs of heavy physical labor and poor health. Although their status of bondage is unknown, these features are consistent with the profile of indentured servants. Although the biomolecular evidence presented here cannot confirm this interpretation, this adds depth to the personal histories of these two men, as many Irish immigrants who arrived in St. Mary’s City (and the Chesapeake region more broadly) during the 17^th^ century came as indentured servants ^22,23^.

By examining patterns of IBD sharing with participants across the US, we learn about the lasting genetic impact of the St. Mary’s founder population on the demography of the country. We observe elevated rates of distant IBD sharing across the southern US, suggesting that the present-day population of the region shares strong genetic similarities to the St. Mary’s founding population. For most distant genetic connections, it is impossible to infer whether participants descend directly from the St. Mary’s individuals, or if both descend from common ancestral populations in Great Britain and Ireland. However, the elevated IBD sharing rate observed among research participants who indicated via survey that they have ancestral ties to 17^th^-century St. Mary’s City suggests that many of these distant genetic connections are likely the result of direct descent from individuals included in this analysis (or their close relatives). Notably, the patterns of sharing that we observed appear similar to the distribution of self-reported “American” ancestry in the 2000 US Census, an identity that is commonly adopted by white Americans whose ancestors immigrated to the Americas many generations ago ^2^. This group may therefore be more likely to trace their ancestry back to one or more colonial-era founder populations, like St. Mary’s City.

We observed strong geographic patterns of IBD sharing among research participants with the closest genetic connections (≥30 cM of shared IBD) to the St. Mary’s individuals. These patterns are consistent with the expected distribution of descendants of the St. Mary’s founder population throughout the US. For instance, we observe a particularly strong concentration of close relatives of the St. Mary’s individuals in Kentucky. This likely reflects a documented migration of Catholic residents from St. Mary’s County (where St. Mary’s City is located) to Kentucky–particularly to Nelson and Washington (formerly Marion) counties–primarily between 1780–1820. Migrating individuals moved in pursuit of economic advantage and religious freedom due to the continued anti-Catholic bias in Maryland during this period ^13–15^. That IBD analysis can detect such a clear signal of this well-documented migration demonstrates the power of this approach.

Finally, we introduced a new framework for re-identifying otherwise nameless historical individuals by analyzing genealogical connections among research participants who share close genetic links to the same historical genomes. This differs from previous identification efforts involving aDNA, which have typically relied on strong prior hypotheses regarding the identity of specific remains. Using this approach, we propose identities for three St. Mary’s individuals: Burial 56 (Leonard Greene), Burial 63 (Anne Cox), and V07C (Governor Thomas Greene). It is highly probable that these three individuals were interred at the Brick Chapel, as the Greene family lived in St. Mary’s City on Greene’s Freehold following Thomas Greene’s time as governor ^17^. While these identifications warrant further verification, the recovery of such a notable figure underscores the limitations of the site’s historical records, as the burial locations of even the most prominent citizens were unrecorded. Conversely, our ability to successfully trace these lineages—while many others remain obscure—likely reflects the high status of the Greene family, which ensured the preservation of genealogical records where others were lost. Therefore, the identification of these individuals as members of the Greene family should be interpreted as the most parsimonious assignment rather than a concrete identification, given the potential for missing branches in self-reported trees. Beyond these specific identifications, our results highlight the highly interconnected nature of colonial founder populations, underscoring the complexity of reconstructing the genetic webs that link present-day people to their historic-era relatives.

We anticipate that this combined genetic and genealogical framework will become increasingly effective at more recent time depths. While this study establishes the method’s viability at ~350 years, applying it to more recent historical cases will likely yield higher identification success rates due to the retention of larger IBD segments among direct descendants (and other close collateral relatives) and the availability of denser genealogical records. Future applications of this framework could be further strengthened by incorporating mitochondrial and Y-chromosome haplogroup data to independently confirm unbroken maternal or paternal lineages. While this orthogonal line of evidence was outside the scope of our initial IRB protocol, its inclusion in future studies could provide an even higher degree of certainty when assigning identities. Our findings therefore serve as a proof of concept for a strategy with broad potential to restore lost identities across history.

By generating genome-wide information from 49 individuals from 17^th^-century St. Mary’s City, Maryland, this study demonstrates the power of genetic analyses to localize the biogeographic origins of colonizing populations and trace the subsequent migrations of their descendants. It also highlights the enduring legacy of this colonial founder population in the present-day US population. This unique case study of a relatively well-documented colonial settlement with a large number of excavated and studied historical human remains illustrates how aDNA can expand our knowledge of founder populations and their connections to contemporary communities. Our findings underscore the potential for future applications of this approach to expand our understanding of migration and ancestry at less-well-documented sites. This approach also has applications outside of the field of genetics, especially for historical archaeologists, bioarchaeologists, and individuals seeking information on their ancestors.

## Resource Availability

### Lead contact

Further information and requests for resources and reagents should be directed to and will be fulfilled by the lead contact, Éadaoin Harney (eadaoinh@23andme.com).

### Materials availability

This study did not generate new unique reagents.

### Data and code availability

- All data needed to evaluate the conclusions in the paper are present in the paper and/or the supplemental information.
- The aligned sequences for the two Calvert individuals and the 46 newly reported St. Mary’s individuals are available from the European Nucleotide Archive under accession numbers PRJEB16735 and PRJEB110565, respectively. Genotype files for pseudo-haploid and phased, imputed versions of the dataset are available at Harvard Dataverse (doi: https://doi.org/10.7910/DVN/6IHEYQ).
- There are restrictions to the availability of 23andMe genotype data due to 23andMe informed consent and privacy guidelines, which result in a contractual obligation of 23andMe to customers to not make data publicly available. However, to ensure replicability of these results, 23andMe agrees that that the publication coauthors will rerun the comparison of historical genomic data against customer genetic data upon request by other academic and nonprofit researchers on reasonable terms to enable the results of the Research Activities to be replicated for at least seven years after publication or for as long as the coauthors are employed by, or otherwise affiliated with 23andMe in a capacity that allows them to rerun the analysis. Wherever possible, supplementary tables are also included that report the summary statistics that were used to create figures that involved 23andMe datasets. Unless another comparable anonymizing approach was specified, these summary statistics were generated with the requirement that in all reported results, any research participant must be indistinguishable from at least four other research participants included in the dataset.
- This paper does not report original code.
- Any additional information required to reanalyze the data reported in this work is available from the lead contact upon request.

## STAR Methods

### Experimental Model and Study Participant Details

#### Community Engagement and Ethics

This study was made possible by the millions of research participants who actively consented to participate in genetic research (including those from 23andMe and others whose data has been released as part of publicly available datasets). Additionally, we recognize the 49 historic-era individuals from Historic St. Mary’s City, who could not directly consent to participate in this study. This research was conducted following all applicable ethical guidelines for the study of historical genomes ^33–35^ and was conducted under the auspices of the HSMC, who were consulted over the course of the study’s design and execution. The skeletal remains from the 49 St. Mary’s City individuals who were sampled as part of this analysis were reinterred with a religious ceremony inside the reconstructed Brick Chapel on September 20^th^, 2025. HSMC has consulted with the descendant community of the early colonists (The Society of the Ark and the Dove) since the beginning of the Chapel project and remains actively engaged in the research effort. Additionally, HSMC shared findings with representatives from several local groups, including the Unified Committee on Afro-American Contributions, National Association for the Advancement of Colored Peoples, and St. Mary’s College of Maryland.

#### Provenance of historical skeletal remains

The skeletons of 65 individuals were documented from excavated single burials within and surrounding the Great Brick Chapel and an ossuary of remains displaced during Chapel construction. Fifty of the 65 individuals were sampled for aDNA analysis as authorized by HSMC, including Philip Calvert, Anne Wolseley Calvert and the Calvert Son. All remains were reinterred at the Chapel site in 2025, in a climate controlled vault within the reconstructed structure and will be accessible for future scientific studies.

#### Present-day research participants

We compared the genomes of the St. Mary’s individuals to data from 11,524,442 research participants who were genotyped by April 26^th^, 2023 by 23andMe Research Institute, a nonprofit genetics and research company. All research participants provided informed consent and answered relevant research questions online via a protocol approved by the external AAHRPP-accredited IRB, Ethical & Independent Review Services (E&I Review), which has since been renamed Salus IRB (https://www.versiticlinicaltrials.org/salusirb). Research participants were included in the analysis on the basis of consent status, which was checked at the time data analyses were initiated. We also included data from the 1000 Genomes Project ^36^ and the People of the British Isles Project ^19^ in these comparisons (Data S7M). When possible, we report results for IBD analyses performed on these datasets in the supplementary materials in order to enable replication of the study results.

We assigned each research participant to a specific geographic location based on either self-reported [1] birth location of all four grandparents (when available and when all four grandparents shared the same birth location) or [2] participant birth location. In cases where participant birth location and grandparental birth location were the same, and where participant birth location provided more fine grained resolution, we favored participant birth location. Otherwise, we favored grandparental birth locations.

We assigned research participants into a variety of cohorts based on their associated location and in some cases, their genetic ancestry, as assigned by the tool Ancestry Composition ^37^. For instance, the US, GB and IE cohorts are comprised of all participants associated with the US, Great Britain and Ireland, respectively, while the European cohort is comprised of all participants associated with the following European countries (as indicated by ISO2 country codes: AL, AD, AM, AT, BY, BE, BA, BG, CH, CY, CZ, DE, DK, EE, ES, FO, FI, FR, GB, GE, GI, GR, HU, HR, IE, IS, IT, LI, LT, LU, LV, MC, MD, ME, MK, MT, NO, NL, PL, PT, RO, RS, RU, SE, SI, SK, SM, TR, UA, VA, and XK) with at least 99% European ancestry.

### Method Details

#### Ancient DNA (aDNA) Sequencing

The remains of Philip Calvert and his infant son were sampled for aDNA in 2015 and the results of this sequencing analysis were described in a report ^8^ that accompanied the public release of their genome-wide data. For all other St. Mary’s individuals, we sampled bone powder from the petrous portion of the temporal bone using a minimally destructive cranial base drilling approach ^38^. Sampling occurred in 2018 and 2022 following curatorial guidelines in place at the time.

aDNA sequencing was performed in dedicated aDNA facilities at Harvard Medical School, which were designed to minimize the likelihood of contamination ^10^. We followed published protocols to extract aDNA from ~37 mg of bone powder ^39^, creating dual-barcoded, double-stranded, partially uracil-DNA glycosylase (UDG) treated libraries ^40,41^. We performed targeted enrichment capture to maximize the proportion of sequences that align to ~1.24 million (1240k) single nucleotide polymorphisms (SNPs) in the nuclear genome in addition to the entire mitochondrial genome ^42–45^. After adding unique indexing barcodes (7 base pairs) to both ends of each DNA molecule, we sequenced the enriched libraries (along with a small amount of the unenriched libraries, to be used for quality assessment) for 2×101 cycles on an Illumina HiSeqX10 or 2×76 cycles on an Illumina NextSeq500 instrument, with 2×7 cycles for indexing.

After sequencing, we used custom software (https://github.com/DReichLab/ADNA-Tools) to trim the molecular adapters and barcodes from the ends of each sequenced read, and then merged paired-end reads–requiring 15 base-pair overlap with no more than three mismatches in low quality bases (<20) or 1 mismatch in high quality bases (≥20). Next, we used samse in BWA (v0.6.1)^25^ to map to the mitochondrial consensus sequence (RSRS) ^46^ and the human reference genome (hg19; https://www.ncbi.nlm.nih.gov/datasets/genome/GCF_000001405.13/). After aligning, we removed duplicates–defined as reads with identical start and end positions, orientation, and barcodes–retaining the highest quality sequence among the duplicates.

To determine whether the sequenced DNA was likely to be authentic to the St. Mary’s individuals, we considered 3 standard criteria for aDNA authenticity. (1) We assessed the cytosine-to-thymine substitution rate at the 5’ end of each molecule, requiring a rate of at least 3% to be considered authentic aDNA. (2) We assessed the mitochondrial match-to-consensus rate, requiring a minimum match rate of 95%. (3) To exclude individuals with evidence of contamination derived from a source of the opposite genetic sex, we required that the number of reads aligning to the Y chromosome divided by the total number of reads aligning to either the X or Y chromosome fall either below 2% (consistent with a genetically female individual) or above 33% (consistent with a genetically male individual) ^47^. (4) For genetically male individuals, we estimated a contamination rate based on the polymorphism rate on the X chromosome (which should have no variation in genetically male individuals who are expected to only have one) and considered a rate over 3% to be indicative of substantial contamination.

We flagged four low-coverage individuals as potentially contaminated based on their mitochondrial match-to-consensus rate. Additionally, we flagged an additional five individuals as having very low coverage (<0.1X). We report individual level results for these samples, but excluded them from all combined analyses of the St. Mary’s individuals (along with all other St. Mary’s individuals with average chromosomal coverage below 1x).

#### Uniparental Haplogroups

For all individuals, we determined mitochondrial haplogroups using haplogrep2 ^26^ and Phylotree version 17, by considering all reads that aligned to the RSRS mitochondrial genome with MAPQ ≥30 and base quality ≥20. For genetically male individuals, we called Y-chromosome haplogroups based on the most derived mutation identified among reads aligning to the Y-chromosome with MAPQ ≥30 and base quality ≥30, using the nomenclature defined by the International Society of Genetic Genealogy (ISOGG) (http://www.isogg.org) version 14.76 (updated April 2019).

#### Publicly available dataset assembly

To compare the St. Mary’s dataset to other publicly available datasets, we created a pseudo-haploid dataset by randomly sampling a single sequence that aligned to each of the 1240k SNP positions targeted during enrichment capture and assigning a diploid genotype based on the allele observed at that position. We merged this pseudo-haploid dataset with data from the Allen Ancient DNA Resource (Version 9) ^36,48–59^ creating two merged datasets with information at 1,233,013 SNPs (the “1240k dataset”) and 584,131 SNPs (the “Human Origins dataset”), respectively.

#### Genetic relatedness

We considered patterns of allele sharing between pairs of individuals to identify 1^st^-3^rd^-degree relative pairs, using the approach introduced in Olalde et al ^48^. Additionally, we considered IBD shared between pairs of individuals (using the approach described below for comparisons of a single St. Mary’s individual to 23andMe research participants) to identify more distant relationships among the St. Mary’s individuals.

#### ADMIXTURE

Using the clustering tool ADMIXTURE^30^ we assigned the ancestry of the St. Mary’s individuals and four present-day populations (YRI.DG, CEU.DG, Pima.DG, ASW.DG) to one or more of three theoretical ancestral populations, which broadly correspond to African, European and Indigenous American ancestry (Figure S1A). Using the 1240k dataset, we pruned SNPs in linkage disequilibrium from the dataset using the parameters --indep-pairwise 200 25 0.4, resulting in a dataset with 462,447 SNPs. Then we ran ten replicates of ADMIXTURE with k=3, and reported the results of the replicate with the highest likelihood.

#### PCA

To understand the biogeographic ancestry of the St. Mary’s individuals, we performed principal components analysis (PCA) using the tool smartpca^29^, projecting the St. Mary’s individuals onto PCA plots generated using the Human Origins dataset with the following parameters: lsqproject:YES and shrinkmode:YES (Figures S1B–D). First, to explore their continental-level ancestry, we projected them onto a PCA generated with 162 populations from around the world. Next to localize the ancestry of the 49 European-related St. Mary’s individuals, we projected them onto a PCA generated with 60 populations from Europe and the Near East. Finally, to assess whether the single African-related St. Mary’s individual had any European or Indigenous American and/or East Asian ancestry, we projected all of the St. Mary’s individuals onto a PCA generated from four populations with West African (YRI.DG), European (CEU.DG), African American (ASW.DG) and East Asian (CHB.DG) ancestry.

#### Imputation

We generated diploid genotype calls at all biallelic SNP positions for all of the St. Mary’s individuals with at least 1x average chromosomal coverage using bcftools mpileup ^25^. We then performed imputation with the tool GLIMPSE (v1.0.0) ^27^, using the phase 3 1000 Genomes reference dataset^37^, filtering out genotype calls with an estimated maximum genotype posterior below 95%. We used EAGLE^28^ to rephase the imputed dataset using a reference panel of either 691,759 research participants genotyped at 454,507 SNPs on the version 1–4 23andMe genotyping platform or 706,995 research participants genotyped at 541,948 SNPs on the version 5 23andMe genotyping platform, with default settings and optional parameters --allowRefAltSwap and --noImpMissing.

#### Identity by Descent

We used the templated positional Burrows–Wheeler transform (TPBWT) IBD detection tool ^31^ to identify IBD segments shared between the imputed St. Mary’s dataset and 23andMe research participants. We used default parameters, including use_phase_correction = True, with L_m = 300 (requiring that matching subsegments span at least 300 SNPs to be considered part of an IBD segment) and missing_site_threshold = 10 (allowing up to 10 consecutive missing SNPs within a segment). While TPBWT outputs segments as short as 3.0 cM by default, we applied minimum length filters to reduce false positives. Specifically, we retained only segments ≥6 cM for individuals with 2–5x average chromosomal coverage, and segments ≥9 cM for individuals with 1–2x coverage (Data S3).^3^

We generated summary statistics that group research participants based on associated location and in some cases, ancestry, to explore geographic patterns of IBD sharing (Data S7C–E, G–I, and Figures S2–S3). To ensure participant anonymity, we rounded the maximum total IBD shared and maximum IBD segment lengths to the nearest digit when IBD sharing is <10 cM, 0 digits when IBD sharing is between 10–30 cM, to the nearest 5 when IBD sharing is between 30–100 cM, and to the nearest 10 when IBD sharing is over 100 cM. All reported averages involve a minimum of 5 participants unless otherwise stated.

For US maps, we report results for participants in the US cohort who reported county-level geographic information (Data S7E). In the European maps, we report results for participants in the European cohort who reported state-level geographic information (Data S7G, Figure S4). To ensure research participant anonymity, we only report coordinate level results for geographic coordinates that have at least 25 associated participants. Since in the majority of cases fewer than five research participants at each coordinate share IBD with one or more of the St. Mary’s individuals, we randomly downsampled the research participant panel and reported results for 80% of research participants, enabling us to report counts fewer than 5 while abiding by 23andMe privacy protection requirements.

In comparisons between pairs of St. Mary’s individuals, we used the lower coverage individual when determining the minimum length threshold (Data S7B). Only comparisons where both individuals have over 1x coverage are shown.

#### IBD Networks

To identify St. Mary’s individuals’ distant and recent connections to genetic groups (Data S7H), we first performed community detection on contemporary participants using planted partition stochastic block models (SBMs) ^24^. SBMs are generative graph models that partition individuals into communities called blocks, based on the total amount of IBD shared with each other with the probability of an edge existing between individuals depending on block membership. We applied this method to individuals who reported all four of their grandparents were born in either Great Britain or Ireland and had ≥99% northern European ancestry (N = 7,872). Individuals included in this cohort were filtered so that no two individuals share ≥ 700 cM. Individuals were clustered with SBMs using a minimum IBD segment length of 5.5 cM and were required to have a ≥ 99% probability of being assigned to a block to be retained. After identifying genetic clusters across Great Britain and Ireland, we determined the average amount of IBD each St. Mary’s individual shares with each of the groups. We used these data to display each St. Mary’s individual’s connections to each group in the form of a graph layout. To achieve this, we first arranged groups within each of the cohorts, using the Force Atlas layout. Force Atlas is an algorithm that situates groups (or nodes) in a graph using a physical “magnetic” model. In this case, groups with more IBD sharing will be attracted to one another and groups with less IBD sharing are repelled. Force Atlas runs until balance between repulsion and attraction is achieved, essentially illustrating the structure of groups via their IBD sharing. After Force Atlas was run on each of the participant cohorts, we independently ran Force Atlas between each St. Mary’s individual and the pre-arranged graph of participants, projecting St. Mary’s individuals onto the structure of each cohort, thus illustrating where St. Mary’s individuals physically fell into the structure of each cohort.

#### Individual-Level Analyses

The subjects of the individual-level pedigree-based analyses provided additional informed consent and shared information about their genealogical connections to 17^th^-century St. Mary’s City online via a protocol approved by the external AAHRPP-accredited IRB, described above. Genealogical information was shared via a secure online folder to which only the participants and the lead author (E.H.) had access. The lead author then used this information to reconstruct genealogical trees for each of the consenting participants, showing lineages of the tree that could be traced to St. Mary’s City and surrounding areas. Publicly available genealogical databases (e.g., FamilySearch.org) were used to validate and add to the trees when possible.

To protect participant privacy, the lead author created masked versions of the pedigrees that excluded personal information–including name, age, sex, and birth and death location–about all individuals included in the trees who were born after 1850. The masked reconstructed pedigrees were then exported from the secure online environment to be used in subsequent analyses and included in publication, in accordance with the IRB approved protocol. To further protect participant privacy, when describing the IBD connections detected between participants whose data were included in these analyses we shared only the number and length of IBD segments shared, while masking the exact genomic location of these segments.

We searched for overlapping names in the reconstructed genealogical trees of participants who shared over 30 cM of IBD to the same sequenced St. Mary’s individuals. Individuals from these parts of the genealogical trees of the same sex as the sequenced individuals were considered to be potential candidates for their identities.

For all candidates, we considered whether the age of the burial (estimated based on the position of the burial, relative to the foundations of the Brick Chapel), grave fill content, shaft orientation, and the age at death of the sequenced individuals (estimated based on morphological analysis) was consistent with the reported date and age of death of the individuals named in the genealogical trees. In cases where a match seemed plausible, we expanded our analysis to also consider whether any of the sequenced individuals had genetic relatives who could be associated with individuals named in the genealogical trees.

### Quantification and Statistical Analysis

#### Randomization Tests

We used randomization tests to determine whether observed patterns of IBD sharing were statistically significant or if they could instead be explained by random chance. For each randomization test, we counted the number of participants who shared a given amount of IBD (either any amount of IBD or at least 30 cM) with one or more of the St. Mary’s individuals from a given region of interest and compared this value to the number of participants with IBD sharing in a random sample of participants that was equal in size to the number of participants from the region of interest (Data S7F). We sampled participants from either the US or European cohorts, restricting the cohorts to participants with at least 99% European ancestry. We performed 1000 replicates of each randomization test and calculated p-values by determining the proportion of replicates where the observed number of participants with IBD sharing exceeded that of the actual region of interest. We considered all p-values <0.01 to be statistically significant, reflecting a Bonferroni correction for the five independent tests performed (0.05/5).

#### Chi Squared and T-Tests

We performed chi square tests to compare the proportion of participants who share IBD with the St. Mary’s individuals between those who answered “Yes” and “No” to the question “Can you trace your relatives back to 17th-century St. Mary’s City, Maryland’s first colonial settlement?” (Data S7J). To ensure these differences were not solely driven by broad population structure, we applied this test to both the full US cohort and a subset restricted to participants with at least 99% European ancestry. Additionally, we used independent t-tests to evaluate differences in the mean amount of shared IBD (in cM) between the two response cohorts. We considered p-values <0.05 to be statistically significant.

## Supplementary Material

1 Data S1. Site Background. Related to Figure 1.

2 Data S2. Genetic Relatedness within the Calvert Family. Related to Figure 1.

3 Data S3. Downsampling analysis. Related to STAR Methods and Table 1.

4 Data S4. Likelihoods for inferring relationships. Related to Figure 5.

5 Data S5. Incorporating historical individuals into modern pedigrees. Related to Figure 5.

6 Data S6. Re-identification of historical individuals. Related to Figure 5.

7

8 Data S7. Tables providing additional information. Related to STAR Methods and Figures 1–5, Table 1, Figures S1-6, and Data S6. (A) St. Mary’s Sample Background (B) Total IBD detected between St. Mary’s Individuals (C) Proportion of 23andMe Participants that Share IBD with St Mary’s Individuals. Cohort membership was determined based on grandparent birth location, or, in cases where all four grandparents were born in different countries or this information was not available, based on participant birth location. *Values are rounded based on the magnitude of IBD sharing as follows: values >100 cM are rounded to the nearest ten, values between 30–100 cM are rounded to the nearest five, values between 10–30 cM are rounded to the nearest integer, and values <10 cM are rounded to one decimal place. **Counts are only reported for comparisons involving all research participants or all historical individuals. Counts are rounded to the nearest 5 and values <=5 are masked. (D) Counts of total IBD shared with one or more St. Mary’s individuals. For all comparisons involving 23andMe participant data (i.e. the 23andMe cohort) counts less than or equal to 5 are masked. Bin sizes were chosen to maximize the number of unmasked counts that could be displayed. (E) IBD between St. Mary’s individuals and research participants at geographic coordinates with at least 25 associated research participants. Values are rounded based on the magnitude of IBD sharing as follows: values >100 cM are rounded to the nearest ten, values between 30–100 cM are rounded to the nearest five, values between 10–30 cM are rounded to the nearest integer, and values <10 cM are rounded to one decimal place. (F) Randomization Tests (G) IBD between St. Mary’s individuals and research participants with ≥99% European ancestry at geographic coordinates with at least 25 associated research participants. Values are rounded based on the magnitude of IBD sharing as follows: values >100 cM are rounded to the nearest ten, values between 30–100 cM are rounded to the nearest five, values between 10–30 cM are rounded to the nearest integer, and values <10 cM are rounded to one decimal place. (H) IBD sharing with genetic clusters from Great Britain and Ireland. (I) IBD sharing with St. Mary’s Individuals in US States. Related to STAR Methods. Counts <=5 are masked. (J) Chi-Squared Test & T-Test. (K) Genealogical information for focal participant 1. (L) Genealogical information for focal participant 2. (M) Individuals from the 1000 Genomes and People of the British Isles (PoBI) datasets included in the 23andMe cohort and used for all IBD analyses. (N) Ancestors and collateral relatives of the 3 focal couples. Individuals whose profiles match that of Burial 56 are shown in green, while partial matches are shown in orange. All other individuals are shaded based on their degree of relationship to one of the focal individuals.

## Figures and Tables

**Table 1.** IBD Sharing between St. Mary’s Individuals and the 23andMe participant panel.

23andMe Participants included in group	No. of 23andMe participa nts in group	Median total IBD in 23andMe participants with IBD detected (cM)*	Maximum total IBD in 23andMe participants with IBD detected (cM)*	Percentage of participants that share IBD with St. Marys individual(s)	Number of participants with any shared IBD**	Number of participants with at least 30 cM IBD**
All Participant	11,524,442	7.3	150	11.85%	1,366,105	9,000
Participants in GB cohort	144,689	7.3	65	24.80%	35,885	60
Participants in IE cohort	11,285	7.6	70	23.70%	2,675	25
Participants in US cohort	2,584,518	7.3	140	15.48%	400,070	2,790
Participants in US cohort with over 99% European ancestry	1,686,066	7.3	140	17.22%	290,345	2,030
Participants in US cohort who answered survey question: Any answer	620,260	7.3	140	16.10%	99,890	780
Participants in US cohort who answered survey question: Yes	14,814	7.5	140	18.54%	2,745	50
Participants in US cohort who answered survey question: No	342,592	7.3	110	14.85%	50,885	380
Participants in US cohort who answered survey question: Not Sure	262,854	7.4	120	17.6%	46,255	350

Cohort membership was determined based on grandparent birth location, or, in cases where all four grandparents were born in different countries or this information was not available, based on participant birth location. We use the ISO2 country code abbreviations to refer to each country (i.e., GB: “Great Britain”, IE: “Ireland”, US: “United States”). The survey question referred to in the final four rows was: “Can you trace your relatives back to 17th-century St. Mary’s City, Maryland’s first colonial settlement?” (possible answers: “Yes”, “No” and “I’m not sure”). To protect the privacy of 23andMe research participants, the following rounding strategies were applied: *Values are rounded based on the magnitude of IBD sharing as follows: values >100 cM are rounded to the nearest ten, values between 30–100 cM are rounded to the nearest five, values between 10–30 cM are rounded to the nearest integer, and values <10 cM are rounded to one decimal place. **Counts are rounded to the nearest 5. (See also Data S3 and S7C)

**Key Resources Table:** 

REAGENT or RESOURCE	SOURCE	IDENTIFIER
Biological samples		
2 Calvert Individuals (Phillip & Son)	Reich et al, 2016 ^8^	N/A
47 newly reported St. Mary's individuals	This paper	N/A
Chemicals, peptides, and recombinant proteins		
23 HI-RPM hybridization buffer	Agilent Technologies	5190-0403
Herculase II Fusion DNA Polymerase	Agilent Technologies	600679
Pfu Turbo Cx Hotstart DNA Polymerase	Agilent Technologies	600412
50% PEG 8000	Anatrace	OPTIMIZE-82 100 ML
0.5 M EDTA pH 8.0	BioExpress	E177
Sera-Mag SpeedBead CarboxylateModified [E3] Magnetic Particles	Cytiva Life Sciences	65152105050250
silica magnetic beads	G-Biosciences	786-916
10 3 T4 RNA Ligase Buffer	New England Biolabs	B0216L
Bst DNA Polymerase2.0, large frag.	New England Biolabs	M0537
UGI	New England Biolabs	M0281
USER enzyme	New England Biolabs	M5505
Buffer PB	QIAGEN	19066
Buffer PE concentrate	QIAGEN	19065
1 M Tris-HCl pH 8.0	Sigma Aldrich	AM9856
1 M NaOH	Sigma Aldrich	71463
20% SDS	Sigma Aldrich	5030
3 M Sodium Acetate (pH 5.2)	Sigma Aldrich	S7899
5 M NaCl	Sigma Aldrich	S5150
Ethanol	Sigma Aldrich	E7023
Guanidine hydrochloride	Sigma Aldrich	G3272
Isopropanol	Sigma Aldrich	650447
PEG-8000	Sigma Aldrich	89510
Proteinase K	Sigma Aldrich	P6556
Tween-20	Sigma Aldrich	P9416
Water	Sigma Aldrich	W4502
103 Buffer Tango	Thermo Fisher Scientific	BY5
503 Denhardt’s solution	Thermo Fisher Scientific	750018
AccuPrime Pfx Polymerase (2.5 U/ul)	Thermo Fisher Scientific	12344032
ATP	Thermo Fisher Scientific	R0441
dNTP Mix	Thermo Fisher Scientific	R1121
Dyna MyOne Streptavidin C1 beads	Thermo Fisher Scientific	65002
FastAP (1 U/mL)	Thermo Fisher Scientific	EF0651
GeneAmp 103 PCR Gold Buffer	Thermo Fisher Scientific	4379874
Human Cot-I DNA	Thermo Fisher Scientific	15279011
Klenow Fragment (10 U/mL)	Thermo Fisher Scientific	EP0052
Maxima Probe qPCR 2xMM	Thermo Fisher Scientific	K0233
Maxima SYBR Green kit	Thermo Fisher Scientific	K0251
Maxima SYBR Green kit	Thermo Fisher Scientific	K0253
Salmon sperm DNA	Thermo Fisher Scientific	15632-011
SSC Buffer (203)	Thermo Fisher Scientific	AM9770
T4 DNA Ligase	Thermo Fisher Scientific	EL0012
T4 DNA Ligase, HC (30U/mL)	Thermo Fisher Scientific	EL0013
T4 DNA Polymerase	Thermo Fisher Scientific	EP0062
T4 Polynucleotide Kinase	Thermo Fisher Scientific	EK0032
23 HI-RPM hybridization buffer	Agilent Technologies	5190-0403
2% Sodium Hypochlorite Solution	Millipore Sigma	Cat# XX0637-76
Acetic Acid, Glacial (TraceMetal Grade)	Fisher Chemical	Cat# A507-P212
7 M HNO3 (Optima)	Fisher Chemical	Cat# A467-2
6 M HCL (TraceMetal Grade)	Fisher Chemical	Cat# A508-4
0.05 M HNO3 (Optima)	Fisher Chemical	Cat# A467-2
30% H2O2 (GR ACS Grade)	Millipore Sigma	Cat# HX0635-2
0.1 M CH3COOH (GR ACS Grade)	Millipore Sigma	Cat# AX0073-6
Critical commercial assays		
HiSeq X Ten Reagent Kit v2.5	Illumina	FC-501 -2521
NextSeq 500/550 High Output Kit v2.5	Illumina	Cat.# 20024906
Deposited data		
Sequencing data from 2 Calvert Individuals (Phillip & Son)	Reich et al, 2016 ^8^	ENA: PRJEB16735
Sequencing data from 47 newly reported St. Mary's individuals	This paper	ENA: PRJEB110565
Genotype data from all 49 St. Mary's individuals (including pseudohaploid genotypes for all individual, and imputed and phased genotypes for the 29 highest coverage individuals)	This paper	Harvard Dataverse: https://doi.org/10.7910/DVN/6IHEYQ
Software and algorithms		
aDNA-Tools	https://github.com/DReichLab/ADNA-Tools	N/A
BWA (v0.6.1)	Li and Durbin, 2009 ^25^	N/A
HaploGrep2	Weissensteiner et al., 2016 ^26^	N/A
GLIMPSE (v1.0.0)	Rubinacci et al., 2021 ^27^	N/A
EAGLE (v2.4.1)	Loh et al, 2016 ^28^	N/A
bcftools (v1.14)	Li and Durbin, 2009 ^25^	N/A
samtools (v1.22)	Li and Durbin, 2009 ^25^	N/A
ADMIXTOOLS (v6)	https://github.com/dReichLab/AdmixTools	N/A
smartpca (v18700)	Patterson et al, 2006 ^29^	N/A
ADMIXTURE (v1.3.0)	Alexander et al, 2009 ^30^	N/A
TPBWT	Freyman et al, 2023 ^31^; https://github.com/23andme/phasedibd	N/A
Bonsai	Jewett et al, 2021 ^32^; https://github.com/23andMe/bonsaitree/tree/main/bonsaitree/v3	N/A

## Highlights

- A novel IBD-based framework for re-identifying anonymous historical remains
- Analysis of 49 St. Mary’s colonists reveals 1.3 million living genetic relatives
- Tracing a founder population’s origins and descendant migrations across the US
- Possible identification of Maryland’s second governor, Thomas Greene, and his family
