# Supplementary material for: The genetic legacy of the 17th-century colonial capital of St. Mary’s City
Source: Curr Biol. Author manuscript; Available in PMC 2026 Aug 19. (PMC13487767; doi:10.1016/j.cub.2026.04.046)
Supplement: Supplementary file 1 [file NIHMS2198057-supplement-1.pdf]

Data S1
Site Background

St. Mary's City was established in 1634 as the founding English settlement of the Maryland colony and one of the earliest in colonial America. Although its role as a capital city was relatively short-lived–in 1695 the capital was moved to Providence (current day Annapolis)–many Americans trace their genealogy back to this historic city [S1].

First envisioned by George Calvert, the first Baron Baltimore, as an expansion of the King's realm and place for religious freedom, especially for persecuted Catholics, a royal charter for the colony of Maryland was granted shortly after Calvert's death in 1632. The first settlers set sail from the Isle of Wight (off the southern coast of England) in November 1633 on the ships The *Ark* and The *Dove*. Led by Leonard Calvert, a son of George Calvert, this original group of settlers was primarily composed of English and some Irish immigrants, of both Catholic and Protestant faiths [S2–4]. Many of the passengers aboard The *Ark* and The *Dove* were indentured servants, including at least one male of mixed African and European (probably Portuguese) descent, named Mathias de Sousa.

After arriving in 1634, settlers founded St. Mary's City on the southern tip of Maryland's western shore near the Chesapeake Bay at the site of an existing Yaocomico village, an Algonquian speaking Native American group. After non-violent negotiations, the Yaocomico willingly left the village to the newly arrived immigrants and the site became the center point of the new colony [S5–8].

Between 1660 and 1661, Philip Calvert (the youngest son of George Calvert) served as the fifth governor of Maryland and afterward held the powerful post of Chancellor. He died in 1683 at the age of 55 or 56. No burial place was recorded. In 1992, bioarchaeological investigations revealed that Philip Calvert was buried alongside his first wife, Anne Wolseley Calvert (died c.1680), and an infant son (born during his second

marriage to Jane Sewell; died 1683). They were interred in lead coffins and buried under the floor of the north transept of the cross-shaped Jesuit Chapel. Due to their unique mortuary treatment, they are the only individuals buried at the Chapel whose identities are believed to be known [S9].

The Chapel was the first brick structure in Maryland (built c. 1667). However, it was preceded by a wooden chapel built in c.1635 that was intentionally burned in a criminal act a decade later. The Brick Chapel, and the surrounding Chapel Field, were a place of burial for nearly a century, becoming the largest 17th-century cemetery in Maryland. In 1704, all worship was forbidden at the Chapel by decree of the Protestant governor and the building was demolished a decade later. Surface traces of the structure and cemetery were obliterated by 1755 when Protestant landowners used the field for agriculture [S10].

Historic St. Mary’s City (HSMC) Commission (the Maryland state museum that preserves and interprets the historical city) confirmed the original location of the Brick Chapel in 1983 and began major archaeological investigations between 1988 and 1996 [S11]. This historically significant symbol of early religious liberty was rebuilt as close to its original form as possible upon the surviving below-ground foundation with completion in 2009. Burials potentially impacted by the reconstruction efforts (a fraction of all of the burials at the site) were excavated [S11]. Human remains excavated from the “Chapel Field” (Site 18ST1-103) have allowed researchers to conduct one of the few large systematic studies of 17th-century skeletons and burial practices in the Chesapeake [S11]. This section of the cemetery includes early immigrants, later colonists, and American-born individuals associated with either the first church or the subsequent Brick Chapel. Based upon archaeological evidence and the documentation of the remains, the burials were dated to three temporal phases: 1) first church, pre-Brick Chapel (1635-c.1667), including a reinterment ossuary pit dating to church construction in 1667, 2) the Brick Chapel (c.1667-1704), and 3) post-Brick Chapel (1704-c.1730). While documents provide the names of a few people who were buried in the Chapel Field, no church burial registers survive and there are no surviving markers, not even for the settlement’s most elite members.
