# Supplementary material for: The genetic legacy of the 17th-century colonial capital of St. Mary’s City
Source: Curr Biol. Author manuscript; Available in PMC 2026 Aug 19. (PMC13487767; doi:10.1016/j.cub.2026.04.046)
Supplement: Supplementary file 2 [file NIHMS2198057-supplement-2.pdf]

Data S2
Genetic Relatedness within the Calvert Family

The prominent British Calvert family played a central role in the founding of the colony of Maryland. George Calvert (1580–1632), held the title of 1st Lord Baltimore. The family's estate, Baltimore Manor, was located in County Longford, Ireland, although he also had homes in London, England and County Wexford, Ireland [S12]. Calvert was a practicing Catholic, and he envisioned Maryland as a haven for English Catholics and others who were facing religious discrimination. While the charter for the new colony was not granted until after his death, his son Cecil (1605–1675), the 2nd Lord Baltimore, organized and financed the colony, sending his brother Leonard (1610–1647) to serve as the first governor. Leonard sailed aboard the two founding ships, Ark and Dove, and established St. Mary's City in 1634. His half-brother Philip (1626–1682), later became the 5th governor of the colony.

Although Philip Calvert is the only one of George Calvert's 12 children whose remains have been definitively identified from the Brick Chapel and Chapel Field cemetery, several members of the extended Calvert family, including Philip's half-brothers Leonard (the first governor) and George (1613–1634), died in St. Mary's during the pre-Brick Chapel period. We therefore considered whether any of the burials genetically related to Philip Calvert could be those of his half-siblings (Data S2 Figure A).

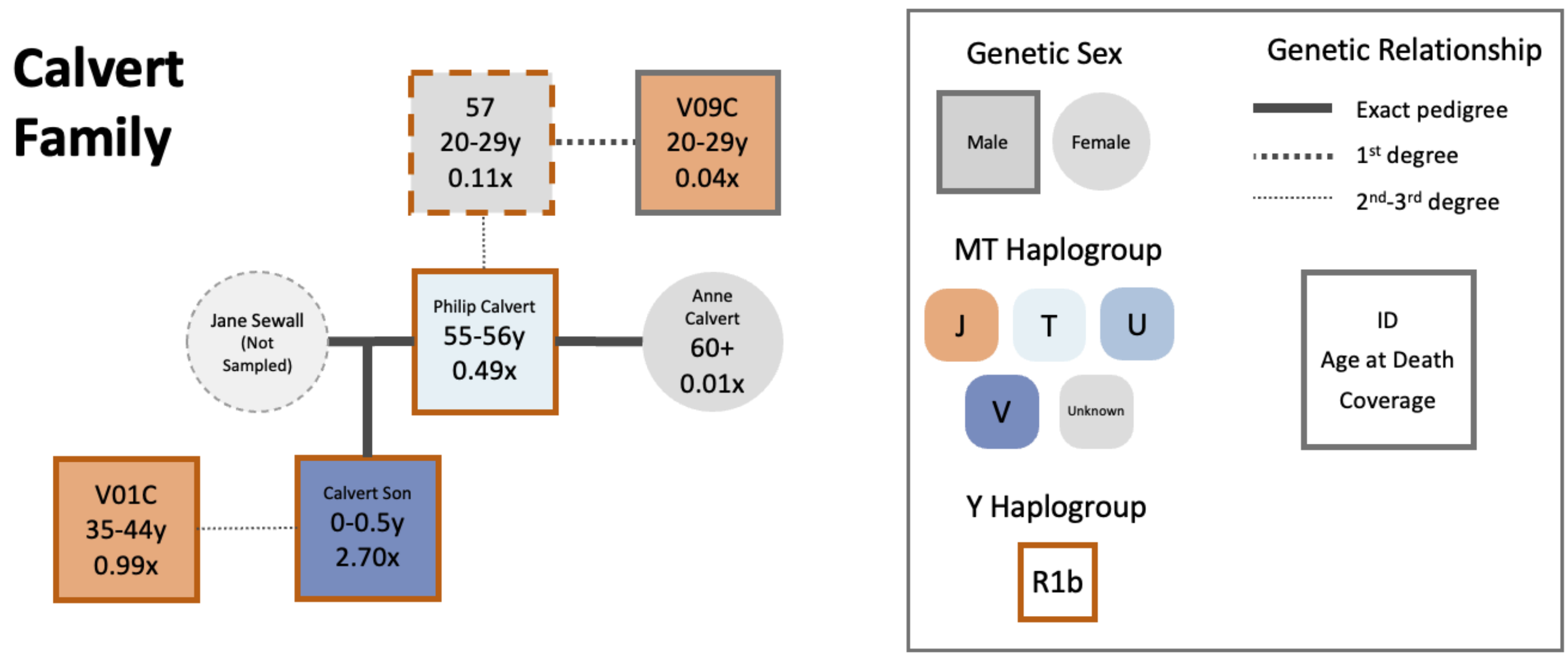

Data S2 Figure A. Genetic kinship and haplogroups of individuals assigned to the Calvert Family. Family groupings were identified among the St. Mary's individuals through genetic connections, except in the case of Anne Wolseley Calvert, who is known from historical records as the first wife of Philip Calvert. Individuals are labeled according to burial ID and average chromosomal coverage. Circular and square markers are used to represent genetically female and male individuals, respectively. The fill color of each marker corresponds to the MT haplogroup, and for genetically male individuals, the outline color corresponds to the Y-chromosome haplogroup. Genetic relationships shared between individuals are indicated by connector lines. A solid dark gray line indicates the exact pedigree, with first degree and second- to third-degree relatives connected by a thick and thin dotted gray line, respectively.

In this study, six individuals comprise "The Calvert Family". They were identified via a combination of historical records, osteological analyses, and genetic connections. This group consists of: Philip Calvert; his first wife (Anne Wolseley Calvert, included based on her documented marriage and identified through her osteological profile and archaeological context, rather than genetic connections); and Philip's infant son, whom it is assumed he fathered with his second wife, Jane Sewell. Her DNA was not analyzed in this study. Three additional individuals share genetic connections to the known Calvert individuals.

The cranial vault, V01C, was interred within the chapel's ossuary, located next to the lead coffin pit in which Philip and his wife and child were buried. The bones from this ossuary represent early interments disturbed when digging the trench for the chapel foundation (c. 1667). V01C shares a genetic connection with the infant

Calvert Son (but not Philip), and is likely a relative of Jane Sewell, Philip's second wife.

We identified Burial 57 (represented by the lower body only due to disturbance of the grave by the construction of the chapel foundation) as a second- or third-degree relative of Philip Calvert. Additionally, we determined that this individual shares a first-degree relationship with V09C, whose cranial vault was also deposited in the ossuary. Although we found no direct genetic link between Philip Calvert and V09C, this result is inconclusive because of poor preservation and the elevated contamination observed in V09C. Further, these factors mean that we cannot exclude the possibility that V09C and Burial 57 represent the same individual.

While V09C could not be assigned a Y-haplogroup, both Burial 57 and Philip Calvert were assigned a Y-chromosome haplogroup belonging to the R1b1a1b1a1a (R-L151) subclade, suggesting that the pair shared a paternal-line connection in the past. However, this haplogroup assignment is broad, and neither individual could be placed into a more specific subclade with confidence; therefore, this paternal-line ancestor may have lived hundreds or thousands of years in the past, so this is not necessarily an indication of recent paternal kinship.

Although precise relationships among these individuals cannot be fully resolved through genetic analysis alone, the presence of multiple genetically related individuals interred in close proximity to the three known Calvert burials is noteworthy. This is significant given that several members of the extended Calvert family, including Philip's half-brothers Leonard (1610–1647) and George (1613–1634), died in St. Mary's during the pre-brick chapel period [S12].

Whether either or both of these burials belong to Philip's half-siblings cannot be discerned with confidence in this analysis, but due to the role that the Calverts played in the founding of the colony of Maryland, this question warrants further study.
