# Supplementary material for: The genetic legacy of the 17th-century colonial capital of St. Mary’s City
Source: Curr Biol. Author manuscript; Available in PMC 2026 Aug 19. (PMC13487767; doi:10.1016/j.cub.2026.04.046)
Supplement: Supplementary file 3 [file NIHMS2198057-supplement-3.pdf]

Data S3
Downsampling analysis

In order to understand how variability in coverage between the historical St. Mary's individuals impacts the amount of IBD sharing detected with present-day research participants, we downsampled all St. Mary's individuals with sufficient coverage to 2x coverage, and then re-imputed and re-ran the IBD analysis. All of the downsampled individuals had a starting coverage of between 2-5x coverage, therefore in the analyses presented in this supplementary data section and in the main text, a 6 cM minimum segment length threshold was applied to all of these individuals. Therefore any differences observed between the results reported in the main text and in this note are due to differences in coverage, not segment length filtering.

We first considered how the distribution of IBD sharing changed between the full coverage and downsampled versions of each individual (Data S3 Figure A). Consistent with the expectation that increased coverage results in a lower false negative rate (as established in [S13], we detect higher rates of IBD sharing in the full coverage version of each sample versus its downsampled counterpart.

Although higher coverage does result in more IBD detected for each of the St. Mary's individuals, the difference in coverage does not appear to be the primary driver of differences in the overall rate of sharing of each historical individual with research participants or of which historical individuals share the longest IBD segments or the most total IBD with research participants. Instead, we found that the relative match rate and maximum amounts of IBD sharing were highly correlated between the full coverage and downsampled versions of each historical individual (Data S3 Figure B).

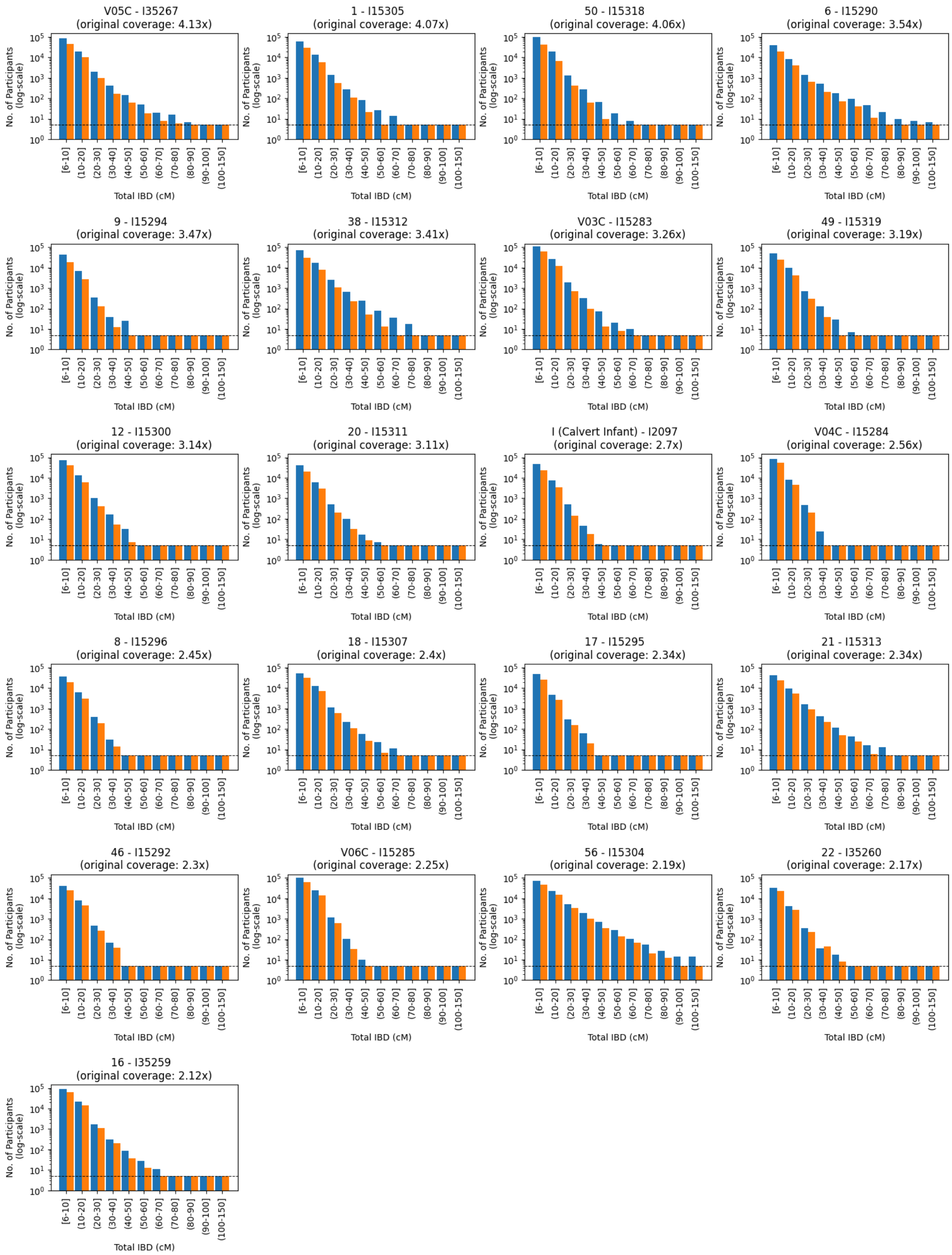

Data S3 Figure A Total IBD sharing with historical St. Mary's individuals for original coverage (blue) and downsampled (orange) datasets. Histograms show the number of research participants that share a given amount of total IBD with each St. Mary's individual. In order to maintain research participant anonymity, bins with five or fewer associated research participants are reported as 5. For both full coverage and downsampled datasets, the minimum IBD segment size considered is 6 cM.

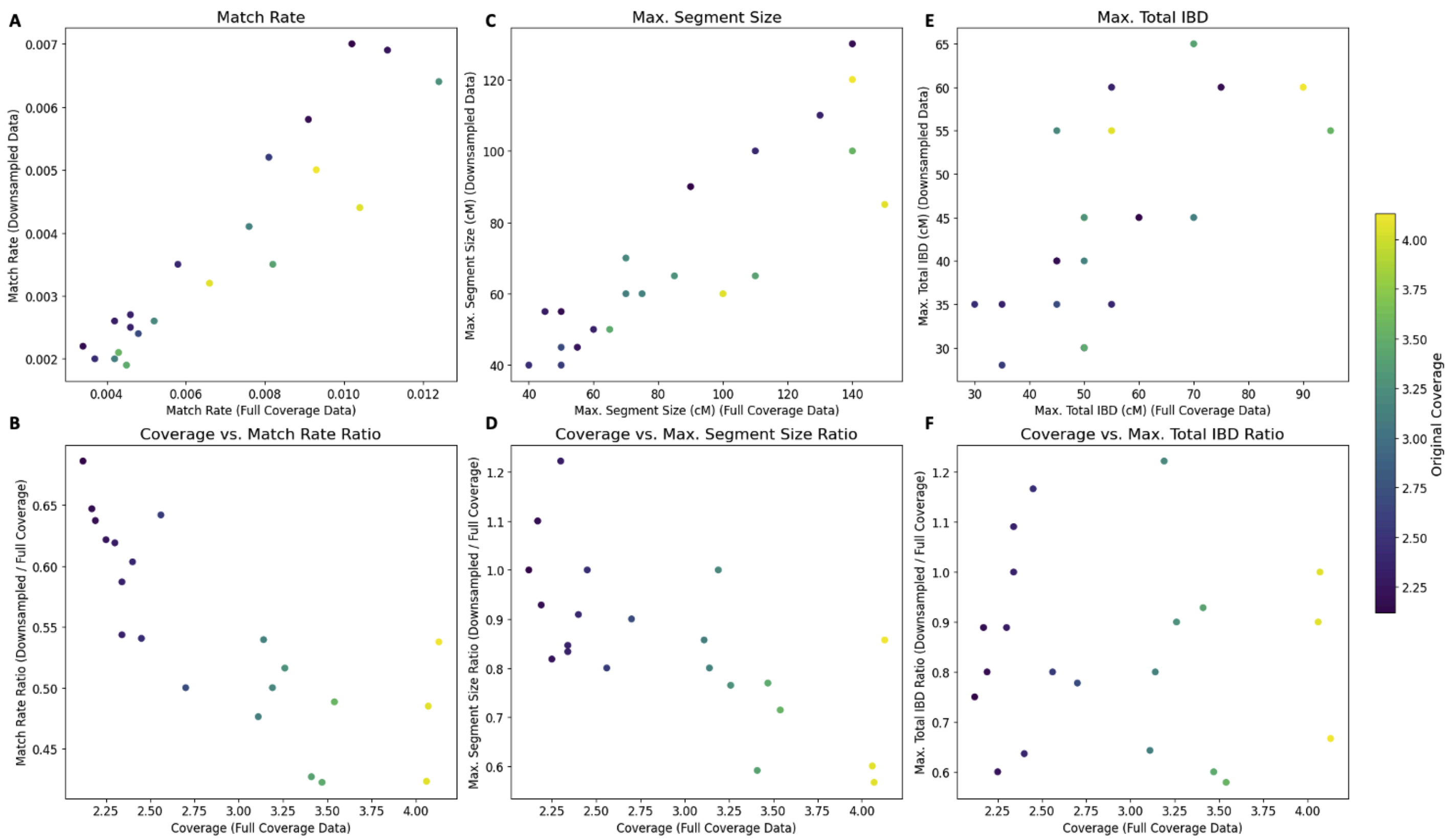

Data S3 Figure B Comparison of IBD sharing metrics between full-coverage and downsampled St. Mary's individuals. The metrics displayed are (A-B) match rate, (C-D) maximum segment size (cM), and (E-F) maximum total IBD (cM). In the top row (A, C, E), the x-axis represents values from full-coverage data, while the y-axis represents values from downsampled data. In the bottom row (B, D, F), the x-axis shows original coverage depth, and the y-axis shows the ratio of downsampled to full-coverage values. All points are colored by coverage. For both full coverage and downsampled datasets, the minimum IBD segment size considered is 6 cM.

These results leave open the possibility that differences in sharing rates reflect real differences in how historical individuals relate to people in the present-day. To explore this possibility further, we examined differences in the geographical distribution of IBD matches in the United States and Europe between the full coverage and downsampled datasets (Data S3 Figure C and Data S3 Figure D). While geographical patterns of sharing were more pronounced in the full coverage dataset, the patterns observed in both the full coverage and downsampled datasets tended to be consistent for each individual. We did not observe any cases where a clear geographic pattern was present at lower coverage but not in the full coverage dataset, suggesting that

the observed geographic patterns are unlikely to be caused by false positive IBD (which we might expect to occur at higher frequencies in lower coverage datasets, particularly if the minimum segment length filtering that had been applied to reduce false positive IBD was insufficient).

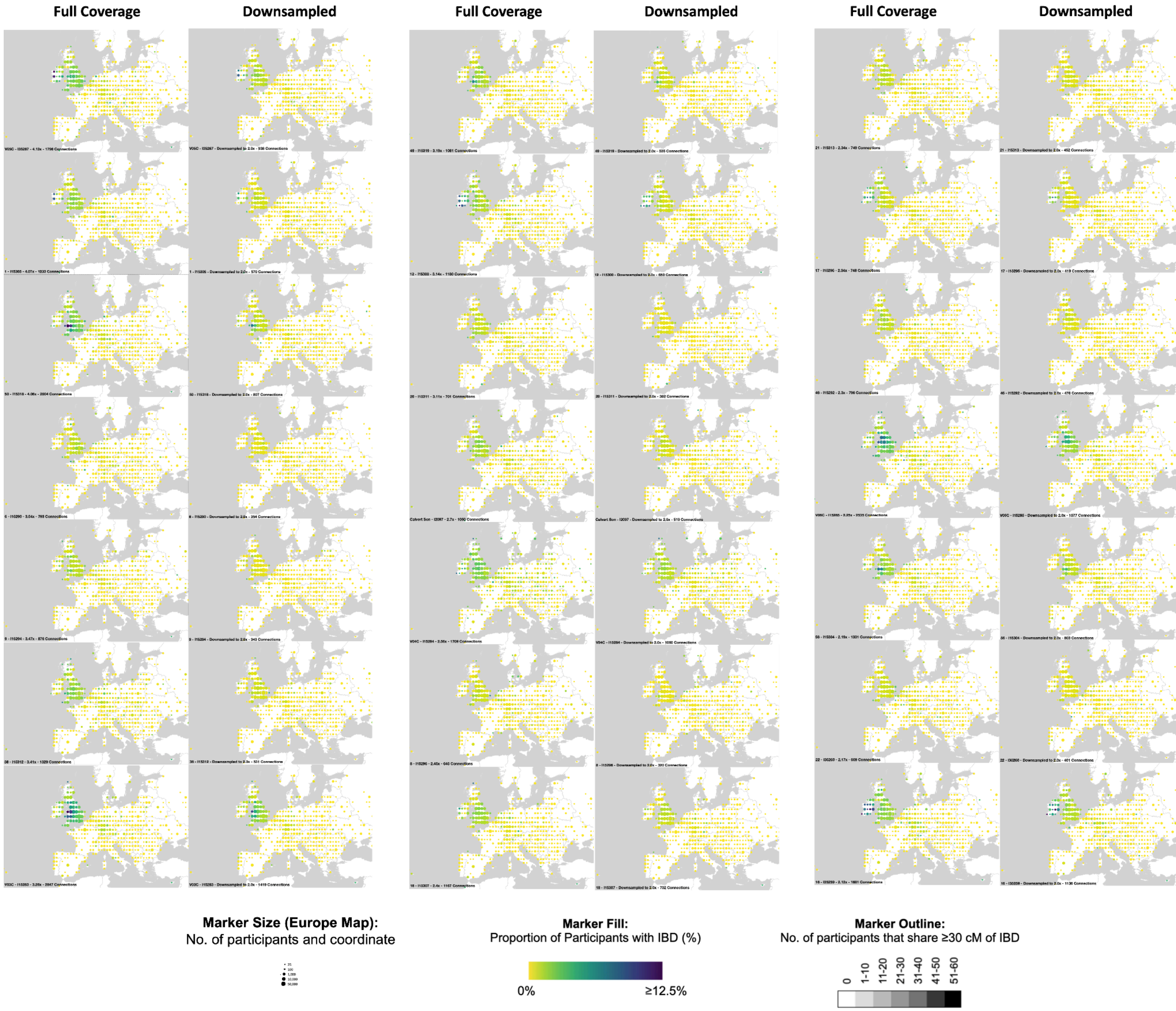

Data S3 Figure C Impact of downsampling on the geographic distribution of IBD sharing with St. Mary's individuals. For each of the St. Mary's individuals with over 2x coverage (one individual per row), we show the proportion (as indicated by the color of the marker) of research participants at each geographic coordinate who share any amount of IBD for analyses performed on the full coverage (left) and downsampled (right) datasets. The size of each marker represents the number of participants at the given geographic coordinate (rounded to the nearest integer). In order to protect participant privacy, we randomly downsampled to include only 80% of participants and only showed results for coordinates with at least 25 associated participants. The total number of participants who share IBD with the St. Mary's individual who are associated with geographic coordinates included in the image is indicated in the figure label, along with the individual ID and their average chromosomal coverage. For each St. Mary's individual we report IBD sharing with research participants with at least 99% European ancestry throughout western and central Europe. The color of the marker outlines indicates the number of participants at each location who share at least 30 cM of IBD with the St. Mary's individual.

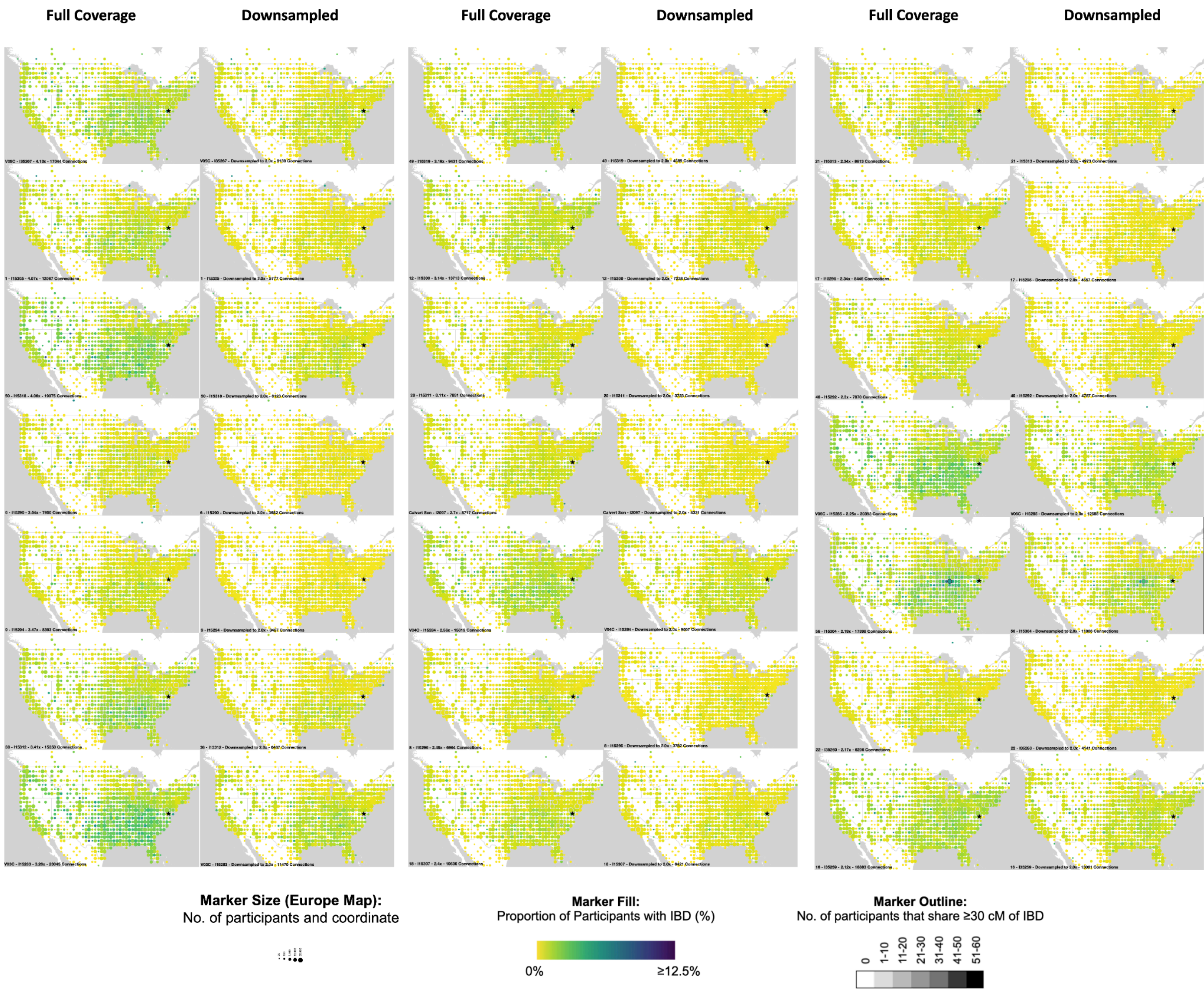

Data S3 Figure D Impact of downsampling on the geographic distribution of IBD sharing with St. Mary's individuals. For each of the St. Mary's individuals with over 2x coverage (one individual per row), we show the proportion (as indicated by the color of the marker) of research participants at each geographic coordinate who share any amount of IBD for analyses performed on the full coverage (left) and downsampled (right) datasets. The size of each marker represents the number of participants at the given geographic coordinate (rounded to the nearest integer). In order to protect participant privacy, we randomly downsampled to include only 80% of participants and only showed results for coordinates with at least 25 associated participants. The total number of participants who share IBD with the St. Mary's individual who are associated with geographic coordinates included in the image is indicated in the figure label, along with the individual ID and their average chromosomal coverage. For each St. Mary's individual we report IBD sharing with research participants in the US. The color of the marker outlines indicates the number of participants at each location who share at least 30 cM of IBD with the St. Mary's individual. The star indicates the location of St. Mary's City, Maryland.

For all individuals, we found that the proportion of matches observed at each geographic coordinate was correlated between the full coverage and downsampled case and in the vast majority of comparisons the match rate was higher in the full coverage case relative to the downsampled case (Data S3 Figure E).

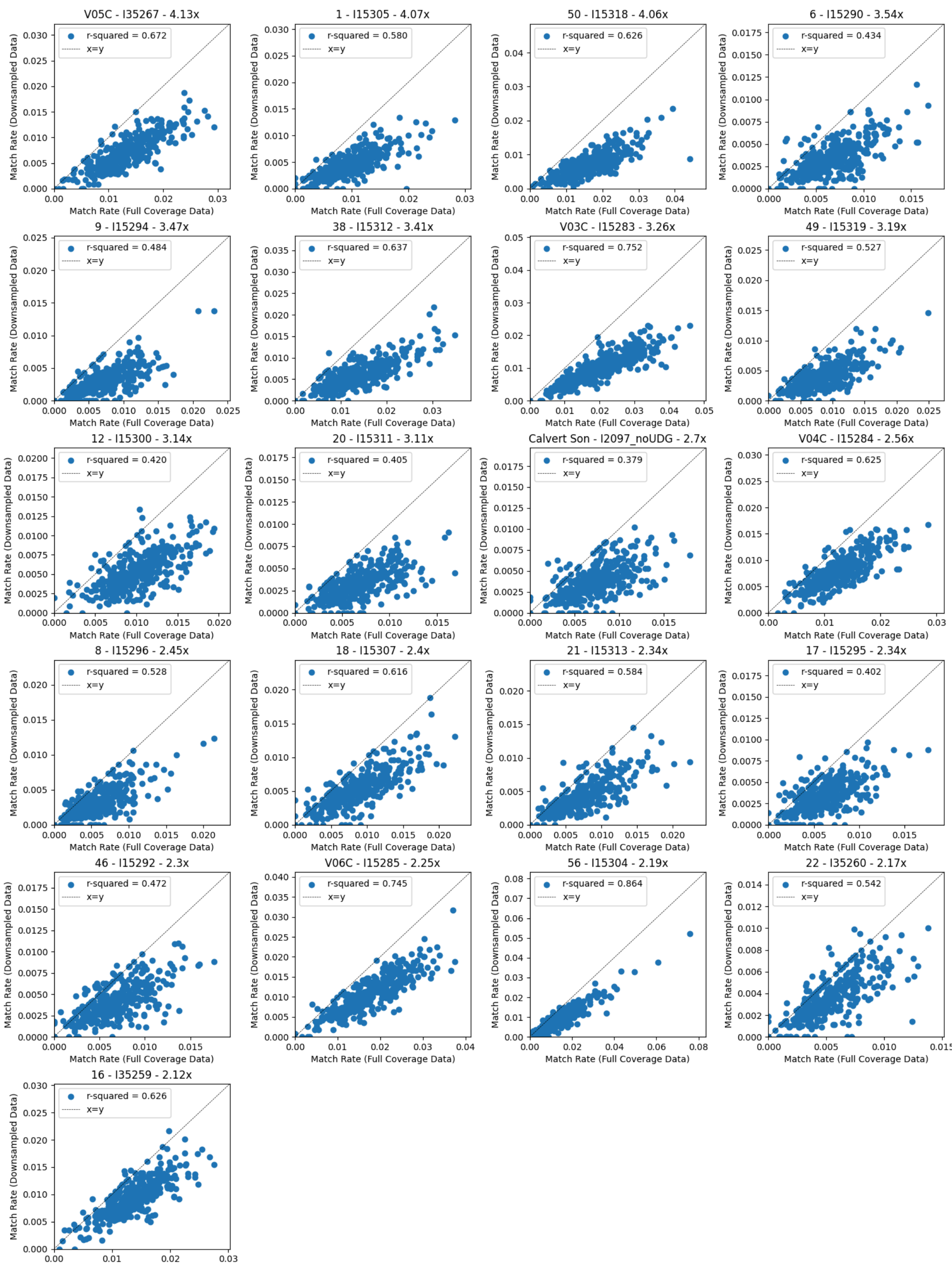

Data S3 Figure E Correlation between proportion of individuals with IBD matches at each geographic coordinate. These plots show the proportion of participants who share IBD with the specified St. Mary's individual at each geographic coordinate that is shown in the maps of Data S3 Figure D (latitude: -23.5 to - 52.5; longitude: -129.5 to -66.5) with at least 500 associated research participants.

Finally, we considered how connections to genetic clusters, like those shown in Figure 3B (replicated in Data S3 Figure FA-B), are impacted by differences in coverage. In Data S3 Figure FC (Data S7H) we show that downsampled individuals experienced a decrease in the number of genetic clusters that they share connections with relative to their full coverage version and that the mean amount of IBD shared with each cluster decreased in the downsampled state. This impacted their position in the IBD network plot, however it was rare for downsampled individuals to gain connections to new genetic clusters relative to their full coverage version (10 instances out of 350 identified clusters). This again suggests that while additional resolution can be gained through increased coverage, matches to genetic clusters in lower coverage individuals (with sufficient coverage to be included in the analysis) can be trusted.

These results suggest that while differences in coverage do contribute to differences in overall match rate between historical and present-day individuals and therefore do impact our ability to identify patterns in genetic sharing, coverage differences are not the sole driver of differences that are observed between historical individuals. Differences in IBD sharing patterns observed across individuals therefore likely reflect real differences in how they relate to present-day populations, perhaps providing evidence of historical lineages that have differing numbers of descendants (although not necessarily direct descendants of the sampled individuals themselves, just the lineages from which they come). However, quantifying differences between these lineages would require an approach that explicitly accounts for coverage differences.

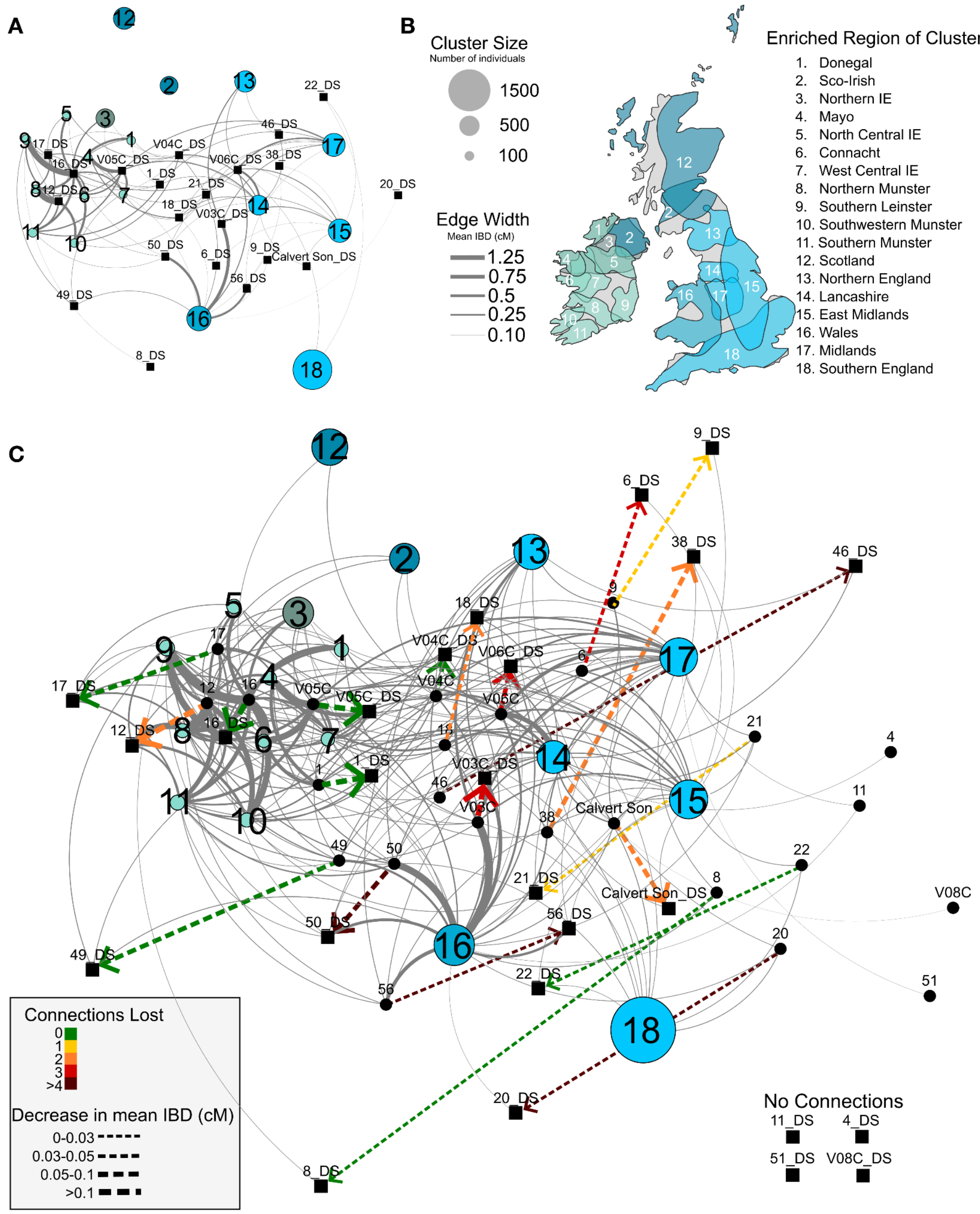

Data S3 Figure F IBD network demonstrating St Mary's individuals' connections to genetic groups in Great Britain and Ireland (N = 7,872). [A] Each circle represents a genetic group that was identified using stochastic block modeling [S14] on IBD connections between individuals that have all 4 of their grandparents born in either Great Britain or Ireland. The size of each genetic group is scaled by the total number of individuals assigned to that (ranging from 88 - 1457). Genetic groups are arranged by the average pairwise IBD sharing between clusters (edges not shown) using a Force Atlas graph layout. Based on the results of their full-coverage genomes, the St Mary's individuals, displayed as squares, are projected over the graph network and arranged based on the average IBD that is shared with each cluster (shown as lines; edges smaller than 0.10 cM are not shown). [B] The geographic distribution of genetic clusters in Great Britain and Ireland. Ranges represent the limits of kernel density estimates based on coordinates of self-reported grandparent locations from individuals in each cluster. [C] Repeats the image shown in panel A, adding squares to represent the position of the St. Mary's individuals after downsampling. The changes in St Mary's individuals' network positions after downsampling are represented by arrows whose color indicate how many genetic group connections are lost by downsampling.
