# Supplementary material for: The genetic legacy of the 17th-century colonial capital of St. Mary’s City
Source: Curr Biol. Author manuscript; Available in PMC 2026 Aug 19. (PMC13487767; doi:10.1016/j.cub.2026.04.046)
Supplement: Supplementary file 4 [file NIHMS2198057-supplement-4.pdf]

Data S4
Likelihoods for inferring relationships

4.1.0 Likelihood

In S[13] we investigated the problem of inferring relationships between high and low coverage individuals. We introduced adjustments to the formulas of [S15] to adjust for the expected number and length of segments shared between two people with a given genealogical relationship when one of those people was sequenced at low coverage.

Let $N_{R,c}$ denote the random variable describing the number of observed segments between a pair of individuals with relationship R when one of the individuals is full coverage and one of the individuals has been sequenced at coverage c. In [S13], we showed that the expected value of $N_{R,c}$ is given by

$$E[N_{R,c}] \ = \ E[N_{R,\infty}]\left[e^{-(u+d)\tau/100} \ - \ \frac{u+d}{u+d+100qc}e^{-(\frac{u+d}{100}+qc)\tau}\right],$$

$$= a2^{1-(u+d)}[r(u\ +\ d)\ +\ c]e^{-(u+d)\tau/100} \times$$

$$\left[e^{-(u+d)\tau/100} \ - \ \frac{u+d}{u+d+100qc}e^{-(\frac{u+d}{100}+qc)\tau}\right] \qquad (1)$$

where

- τ is the smallest observable segment length in cM,
- c is the number of autosomes ($c\ =\ 22$ in humans),
- u is the number of meioses “up” from the first individual to their common ancestor with the second individual,

- d is the number of meioses down from the common ancestor to the second individual,
- r is the expected number of cross-over events per meiosis ($r \approx 35$ in humans),
- a is the number of common ancestors ($a = 1$ or 2),
- $R = (u, d, a)$, and
- q is an empirically determined constant (where $q \approx 0.179$) .

We also showed that the expected length $L_{R,c}$ of a segment shared between two people with relationship R, one of whom has full coverage and the other of whom has low coverage c can be expressed as

$$E[L_{R,c}] = \tau + \left(E[L_{R,\infty}] - \tau\right)\left(1 - e^{-pc}\right)$$

$$= \tau + \left(\frac{100}{u+d} - \tau\right)\left(1 - e^{-pc}\right), \quad (2)$$

where $p \approx 1.79$.

In [S16], we showed that in order to properly infer the relationship between a pair of individuals who are observed to share at least one segment of IBD in the first place, it is necessary to condition on the event that IBD was observed at all. The expression for the probability density of a set of observed segments with given lengths, given that at least one segment was observed, is given by Equation (9) of [S16]. Substituting Equations (1) and (2) into that equation instead of the expected mean number and mean length found there, we obtain

$$Pr(l_1, l_2, \ldots, l_n \mid R, c) \approx \frac{1}{1-e^{-\eta-E[N_{R,c}]}} \sum_{i=1}^{n} \left[\prod_{j=1}^{i} E[L_{R,c}] e^{-E[L_{R,c}](l^{(i)}-\tau)} \right] \frac{E[N_{R,c}] e^{-E[N_{R,c}]}}{i!} \left[\prod_{j=i+1}^{n} \lambda e^{-\lambda(l^{(j)}-\tau)} \right] \frac{\eta^{n-i} e^{-\eta}}{(n-i)!}, \quad (3)$$

where

- $l^{(i)}$ is the ith longest observed IBD segment between the pair of putative relatives,
- η is the expected number of false positive IBD segments observed between the pair, and

- λ is one over the expected length of a false positive IBD segment.

The false positive segments include both false positives due to low coverage and also false positives due to background IBD. Thus, η and λ reflect both of these kinds of segments.

We found in [S13] that the expected number of false positive IBD segments due to coverage is

$$\eta \approx \gamma_1/c,$$

and

$$\lambda \approx c/\gamma_2$$

where $\gamma_1 \approx 0.036$ and $\gamma_2 \approx 0.141$. Note that we actually found that the total length of false positive background IBD had mean $E[T] \approx \gamma_2/c$, but since individuals with coverage above 1 have a false positive IBD segment only about 4% of the time, the total length of false positive IBD typically comes from just one segment. Thus, we make the approximations $\lambda \approx c/\gamma_2$ and $\gamma_2 \approx 0.141$.

In most populations, the expected number of background IBD segments per pair is approximately 0.23 and these segments have a length of 2 cM beyond the minimum segment length on average. Thus, the background IBD in a population typically dramatically overshadows the false positive IBD arising from low coverage, both in the number and lengths of segments. So, for our analyses here, we set

$$\eta = 0.23$$

and

$$\lambda = 1/2.$$

As in [S13], we model the difference in ages δ_R between a pair of individuals separated by relationship R as a Gaussian random variable with mean given by $\mu_{\delta_R} = (d - u)\hat{\mu}_{pc}$ and variance $\sigma^2_{\delta_R} = (u + d)\sigma^2_{pc}$, where $\hat{\mu}_{pc}$ and σ^2_{pc} are the empirically measured mean and variance of the age difference between a parent and a child. This gives

$$f(\delta;\ R) = \frac{1}{\sqrt{2\pi\sigma^2_{\delta_R}}} e^{-\frac{(\delta-\mu_{\delta_R})^2}{2\sigma^2_{\delta_R}}}. \quad (4)$$

All together, we find that the likelihood of relationship R, given the observed segment lengths and the difference in ages between the individuals is

$$Pr(l_1, l_2, \ldots, l_n, \delta \mid R, c) \approx \frac{1}{1-e^{-\eta-E[N_{R,c}]}} \sum_{i=1}^{n} \left[\prod_{j=1}^{i} E[L_{R,c}] e^{-E[L_{R,c}](l^{(i)}-\tau)} \right] \frac{E[N_{R,c}] e^{-E[N_{R,c}]}}{i!} \left[\prod_{j=i+1}^{n} \lambda e^{-\lambda(l^{(j)}-\tau)} \right] \frac{\eta^{n-i} e^{-\eta}}{(n-i)!} f(\delta|R), \quad (5)$$

where δ is the observed age difference between the two people.

4.1.1 Prior

To obtain the probability of a particular relationship R, given the observed IBD, we may wish to use a prior and infer the relationship within a Bayesian context if we are sufficiently confident in that prior.

The prior or R is the relative fraction of IBD-sharing relationships of type R in the population. Here, we note that we are specifically interested in IBD-sharing relationships because we condition on observing IBD when computing our relationship estimate.

To find the expected number of relatives of type R, regardless of whether they share IBD, note that a person has 2 parents, 4 grandparents and 2^u ancestors overall at generation u in the past. Assuming that each couple has f offspring on average who survive to adulthood to reproduce, we find that each ancestral couple has f^d descendants, d generations in the future. We can use these results to obtain the expected number of relatives of each type R.

Note that although each person has 2^g ancestors g generations ago, not all of these ancestors are distinct because the population has a finite bounded size of N diploid individuals. Moreover, although each couple has f^g descendants on average, again not all of these individuals are distinct because the population size is finite. Let a_u denote the number of distinct ancestors u generations ago and let s_d denote the number of descendants each couple has d generations in the future. [S16] showed that the expected number of distinct ancestors can be found using the recursion

$$E[a_g|a_{g-1}] = N\left[1 - (1 - \tfrac{1}{N})^{2a_{g-1}}\right], \tag{6}$$

and by the same token, the expected number of distinct descendants per couple is given by the recursion

$$E[s_g|s_{g-1}] = N\left[1 - (1 - \tfrac{1}{N})^{fs_{g-1}}\right]. \tag{7}$$

By applying Recursion (6) u times and by applying Recursion (7) d times, we can find the expected number of distinct relatives of type $R = (u, d, a)$. Specifically, we have

$$E[a_u] \approx << E[a_g|a_{g-1}];\ E[a_0] = 1; u >>, \tag{8}$$

and

$$E[R] \approx << E[s_g | s_{g-1}];\ E[s_0] = E[a_u]/2; d >>, \quad (9)$$

where $<< R(\bullet);\ cond; n >>$ denotes the outcome of applying recurrence relation $R(\bullet)$, n times with starting condition $cond$.

Equation (9) is the number of distinct relatives, but not all of these relatives share IBD with the focal person. The probability that a pair of genealogical relatives of type R share any detectable IBD at all comes down to the probability that two people separated by $|u - d|$ generations share any detectable IBD at all. Again, we can use results from [S16] to obtain this probability.

Let $q = |u - d|$ and consider the probability that two relatives A and B share IBD through a relative who lived g generations before the more ancient one. For simplicity, we'll assume that B is the more ancient relative so that $d < u$. We will count generations g starting from the generation in which B lived, so that $R = (u, d, a) = (q + g, g, a)$.

Now, as we noted in [S16], each parental copy of the genome of individual A came from $r(q + g) + c$ segments g generations in the past. This result comes from an analogous argument to that of [S17] and it is due to the fact that there are $r(q + g)$ recombination events by $q + g$ generations in the past and there are $c - 1$ additional breakpoints between the c independently assorting autosomes. This leads to $r(q + g) + c$ segments $q + g$ generations ago. Similarly, each parental copy of the genome of individual B came from $rg + c$ segments g generations in the past.

Let $\lambda_{A,g}$ denote the parameter of the exponential distribution describing the lengths of the segments ancestral to A in generation g in the past and let $\lambda_{B,g}$ be the parameter of the exponential distribution describing the lengths of segments ancestral to B in generation g. If we consider how these segments spatially overlap one

another without considering whether or not they came from the same ancestral individual, we find that the length of each overlap is exponentially distributed with parameter $\lambda_{A,g} + \lambda_{B,g}$, since it is the distribution of the minimum of these lengths. It follows that the number of such overlapping segments is $L_{genome}/(\lambda_{A,g} + \lambda_{B,g})$, where L_{genome} is the length of one parental copy of the genome in cM.

From coalescent theory, the probability that a given one of these segments finds its common ancestor in generation g is $\frac{1}{2N}e^{-g/2N}$ and the probability that it is longer than τ cM is $e^{-\tau\frac{2g+q}{100}}$. Putting all this together, we find that the expected number of detectable segments inherited g generations in the past is

$$E[S_{R,g}] \approx 4\,\frac{L_{genome}}{\lambda_{A,g}+\lambda_{B,g}}\,\frac{1}{2N}\,e^{-g/2N}\,e^{-\tau\frac{2g+1}{100}}, \tag{10}$$

where the factor of 4 comes from the fact that A and B each has two copies of the genome.

Summing Equation (10) over all generations g, we find that the expected number of shared segments between A and B is

$$E[S_R] \approx \sum_{g=0}^{\infty} 4\,\frac{L_{genome}}{\lambda_{A,g}+\lambda_{B,g}}\,\frac{1}{2N}\,e^{-g/2N}\,e^{-\tau\frac{2g+1}{100}}. \tag{11}$$

Thus, the probability that A and B share an observable segment of IBD is

$$Pr(O_R) \approx 1 - e^{-E[S_R]}. \tag{12}$$

Putting together Equations (9) and (12), we find that the expected number of IBD-sharing relatives of type R is

$$E[R|O] = E[R]\, Pr(O_R) = \ll E[s_g|s_{g-1}];\ E[s_0] = E[a_u]/2; d \gg \left(1 - e^{-E[S_R]}\right), \quad (13)$$

where $E[a_u]$ comes from Equation (8). Computing $E[R|O]$ for a wide range of relationship types and normalizing their counts gives the prior probability of observing an IBD-sharing relationship of type R:

$$Pr(R|O) = E[R|O] \,/\, \sum_{R'} E[R'|O]. \quad (14)$$

Combining Equations (5) and (14) gives the posterior probability of observing a relationship of type R, conditional on a set of observed segment lengths.

4.2.0 How much shared IBD indicates a direct ancestor?

It is of interest to infer whether the sampled historical individuals are the direct ancestors of their modern-day relatives or if they are collateral relatives. To answer this question, we computed the posterior probability of many different relationships for two individuals separated in age by 300 years and investigated which numbers and lengths of segments corresponded to inferred direct ancestors.

Data S4 Figure A shows the posterior probability of various relationship types for various numbers of segments and total lengths of IBD. From Data S4 Figure A , we can see that for one segment ($n = 1$) of length $L = 30$ cM, the most probable relationship type is a 14th degree relationship. Moreover, since the posterior curve for the case of 14 degrees is concave (i.e.., not monotonically decreasing from the direct ancestral relationship to the other relationships), the most likely relationship is not a direct ancestral relationship. For $n = 2$ segments

and a total length of $L = 30$ cM, the most likely relationship is 15th degree and, again, it is not a direct ancestral relationship. The same is true for $L = 30$ cM and $n = 3$.

However, from Data S4 Figure A , we see that for $L = 50$ and $n = 1$ or $n = 2$, both of the most likely relationships are direct ancestral relationships since the curves for the most likely relationships take on their maximum values at direct ancestral relationships. And for all greater values of L, the most likely relationship is a direct ancestral relationship for all values of n.

The posterior density is rather flat; however, the direct ancestral relationship is often quite a bit more likely than the next most likely relationship. This can be seen by comparing the numerical value for the maximum likelihood between curves in the legend.

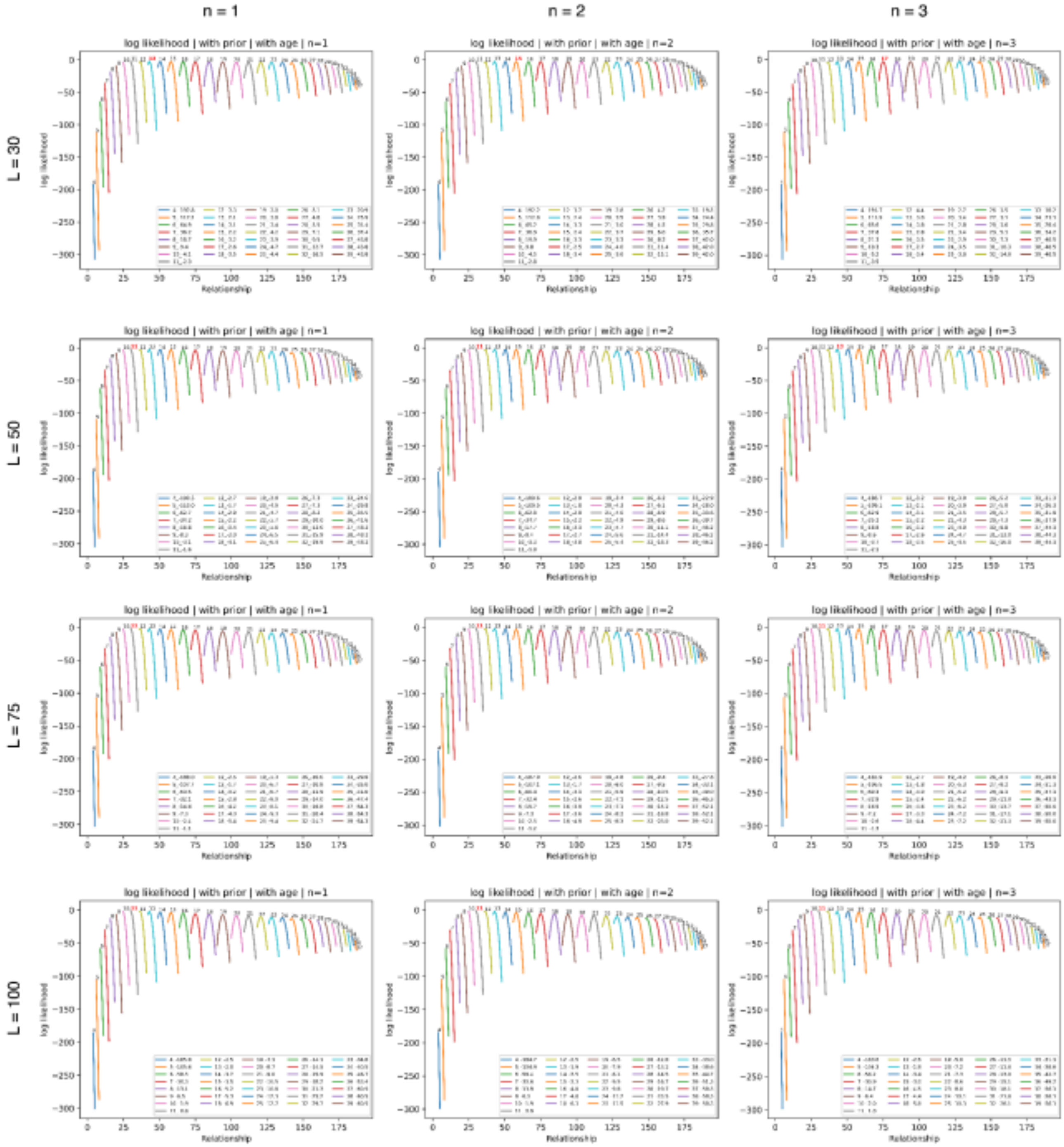

Data S4 Figure A: The posterior probability of relationships of different types, for various numbers of segments n and total lengths L. Rows correspond to total amounts of observed IBD in cM and columns correspond to the number of observed segments. Within each cell, relationships *R*=(*u*, *d*, *a*) are first ordered by degree (*u*+*d*-*a*+1), then by u for all values of u satisfying *u* ≤ *d.* Curves are colored by degree and the degree of the curve is shown above it and in the legend. The ordering of relationships R within each degree implies that the left-most relationship is directly ancestral (*u* = 0). When the curve is strictly decreasing, the most likely relationship is a direct ancestral one. The most likely degree is shown in bold red above the corresponding curve. The legend shows the degree and the log likelihood.

4.3.0 Attaching historical people to modern pedigrees

We used the algorithm Bonsai [S18] to connect historical sequenced individuals from Historic St. Mary's City to modern study participants. For each participant in our analysis, we first inferred a pedigree for them by finding all relatives sharing at least 100 cM with the study participant. We then computed IBD between all pairs of relatives using our in-house unphased IBD algorithm [S19] which is very similar to the IBIS algorithm of [S20]. Finally, we inferred the pedigree using Bonsai with default parameters. We then inferred phased IBD between each member of the pedigree and the historical individual we wanted to attach using the PhasedIBD algorithm of [S21]. We used unphased IBD for inferring the focal pedigree and phased IBD for attaching the historical individual because unphased IBD is more accurate for close relationships whereas phased IBD is much more accurate for distant relationships [S21].

Bonsai was modified to implement the likelihood in Equation (3) and, optionally, the prior in Equation (14). Equation (3) has two forms: a form that conditions on the event that IBD is observed in the first place between a pair of individuals and a form that does not condition on the event that IBD is observed [S16]. The conditional likelihood was applied to each pair of individuals that was ascertained because they shared IBD and the unconditional likelihood was applied to pairs that were not so ascertained. In practice, every pair that included the study participant, including the pair composed of the study participant and the historical individual, was computed using the conditional density, whereas every other pair was computed using the unconditional density.

4.3.1 IBD sharing levels consistent with direct ancestors in the pedigree analysis

We presented an analysis of the length of IBD that is consistent with a direct ancestral relationship in Section 4.2.0. In the case of the pedigree analysis, we have a further piece of information from the genealogical record, which is that our study participants had one or more ancestors who lived in Historic St. Mary's City. Typically,

each study participant had several such ancestors. This fact imposes further constraints on the prior probability that a randomly chosen person from this sample is a relative of a given type R. In particular, the number of direct ancestors is elevated in the sample compared with a uniformly random draw from the population. Thus, the correct prior distribution for this sample is different from the prior distribution in Data S4 Figure A.

Let H be the total number of historical individuals who lived in Historic St. Mary's City. We know that each of our study participants had at last one direct ancestor among the H historical individuals, and they typically had many direct ancestors. Thus, the relative proportions of direct ancestors and their descendants must be correspondingly re-normalized in the prior to account for the fact direct ancestors made up a fraction of at least $1/H$ in the sampled population. The exact renormalization depends on assumptions about the historical population, but suffice it to say that even if each study participant had only a single ancestor in the set of H historical individuals from Historic St. Mary's City, the relative probability of a direct ancestor or their descendants ($\geq 1/H$) would be orders of magnitude higher than the renormalized probabilities of collateral relatives of direct ancestors, which are on the order of 10^{-6} even before renormalization. Thus, if we infer a direct ancestral relationship in our pedigree analysis (which we do), we can be relatively confident that the relationship is indeed directly ancestral.
