# Supplementary material for: The genetic legacy of the 17th-century colonial capital of St. Mary’s City
Source: Curr Biol. Author manuscript; Available in PMC 2026 Aug 19. (PMC13487767; doi:10.1016/j.cub.2026.04.046)
Supplement: Supplementary file 5 [file NIHMS2198057-supplement-5.pdf]

Data S5

Incorporating historical individuals into modern pedigrees

To give name to the historical individuals from St. Mary's City, we compared self-reported genealogical trees with pedigrees inferred from genetic data. If the inferred location in a genetically inferred pedigree of a historical individual corresponded with the location of a named individual in the genealogy, the named individual could be a match for the historical individual.

A major challenge of this approach is that the self-reported pedigrees of most focal participants contain multiple lineages connecting them with ancestors from St. Mary's City. These lineages are typically not genetically distinguishable from one another. For example, in Data S4 Figure A, suppose we know that the historical individual (green) shares IBD with the focal participant (shown in purple) and to other present-day 23andMe research participants (shown in blue). Thus, this ancestor is more likely to come from the lineage that is ancestral to both the purple and blue individuals (shown in yellow) than from any of the other lineages (shown in grey or orange) because only yellow nodes are genealogically related to all genotyped nodes. However, within the yellow group, each node in a given generation has the same expected IBD sharing pattern with the genotyped individuals. As a result, although we can potentially narrow down the location of the historical individual to the yellow cluster and a particular generation (or range of generations), we can't narrow down which individual in that generation corresponds to the historical person.

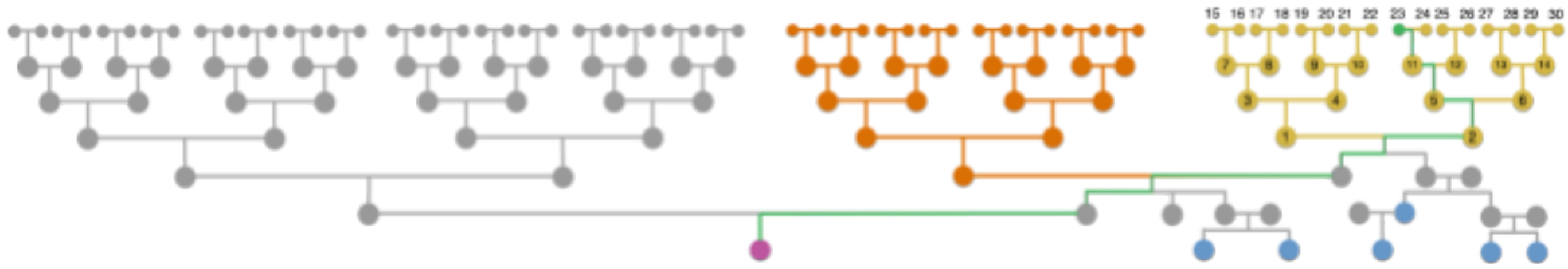

Data S5 Figure A: The challenge of matching genetically inferred pedigrees with self-reported genealogies. A survey respondent is shown in magenta. Their relatives who are genotyped in the 23andMe database and who are consented for 23andMe research are shown in blue. Their self-reported relatives from Historic St. Mary's City are shown in orange and yellow. The genetically inferred position of a sampled individual from Historic St. Mary's City is shown in green and the path from the survey respondent to the placed historical individual is also shown in green.

This problem of alignment between genetic and genealogical pedigrees is exacerbated in our sample by the fact that the historical individuals lived approximately ten generations before the focal participants, yielding up to 2^{10} indistinguishable ancestors, depending on the number of ancestors the focal participant had from Historic St. Mary's City.

The problem is effectively one of triangulation. In the pedigree in Data S5 Figure A, if the historical individual's generation were inferred three generations before the generation of the focal individual, then we would only have two potential matches: nodes 1 and 2. The genetically inferred sex of the individual would then be enough to re-identify them. Similarly, if a genotyped descendant of node 23 (shown in green) existed in the pedigree who was related to the historical individual through a child of 23 other than node 11, that would provide evidence that node 23 is a good match for the historical individual.

Burial 56

In one case we were able to combine survey pedigrees to achieve a deep triangulation. Burial 56 was related to two present-day focal participants, sharing over 30 cM of IBD with each (Data S5 Table A). This fact allowed us to infer the placement of Burial 56 in the pedigree of each of the two survey participants using age and genotype data alone. The placement is found by examining all possible ways in which the historical person can be placed in a modern pedigree. We place the historical relative in a position such that the pairwise relationship to each genotyped pedigree member is most consistent with the IBD shared with each genotyped pedigree

member, and the point estimated age difference between the historical individual and each genotyped individual is most consistent with their pairwise relationship.

The most likely position of Burial 56 in the pedigrees of the two research participants is shown in Figure 5C. In Figure 5C, we have drawn both participant pedigrees together, linked by the individual from Burial 56.

Data S5 Table A. Summary of genetic and genealogical sharing in the pedigree related to Burial 56.

Number of genotyped nodes	37
Number of genotyped relatives sharing at least one detectable segment with Burial 56	7
Mean total IBD shared between genotyped individuals and Burial 56, given that the individual shares at least one segment.	26.8
Mean inferred degree between a genotyped individual and Burial 56	10.8
Number of individuals inferred to be genetically related to Burial 56	20
Total cM shared between Participant 1 and burial 56	48 cM
Total cM shared between Participant 2 and burial 56	33 cM
Number of segments shared between Participant 1 and burial 56	2
Number of segments shared between Participant 2 and burial 56	1

From the pedigree in Figure 5C, we are able to narrow down the set of potential ancestral branches on which Burial 56 can be found and we were able to estimate the approximate generation in which they lived. The information gained from this analysis was compared with the self-reported genealogical pedigree, yielding a small set of potential candidates for reidentification (See Data S4).
