# Supplementary material for: The genetic legacy of the 17th-century colonial capital of St. Mary’s City
Source: Curr Biol. Author manuscript; Available in PMC 2026 Aug 19. (PMC13487767; doi:10.1016/j.cub.2026.04.046)
Supplement: Supplementary file 6 [file NIHMS2198057-supplement-6.pdf]

Data S6

Re-identification of historical individuals

We explored whether the known genealogical histories of study participants could be combined with genetically inferred pedigrees to re-identify any of the sequenced historical individuals from St. Mary's City. As a test case, we collected detailed genealogical histories from consenting research participants who could trace their ancestral lineages back to one or more individuals in Historic St. Mary's City. These genealogical histories could be compared with the genetically inferred pedigrees. Whenever possible, we filled in missing information on the participant-provided genealogical trees using publicly available records obtained from FamilySearch (FamilySearch.com).

In general, it is difficult to match individuals between a genetic and genealogical pedigree because of the inherent uncertainty in genetically inferred pedigrees and the incompleteness of both genealogical and genetic family trees. Sometimes, study participants were related to the same historical individual through multiple ancestral lineages. Thus, it was generally prohibitively challenging to match any particular genealogically recorded ancestor to any particular node in a genetically inferred pedigree.

However, in one instance, a sequenced historical individual (Burial 56) shared over 30 cM of IBD with two different present-day study participants (48 cM with Study Participant 1 and 33 cM with Study Participant 2), suggesting that the participants were relatively recent descendants or collateral relatives of that historical individual. Our genetic pedigree analysis predicted Burial 56 to be a direct ancestor of both study participants, separated by 11 degrees for participant 1 and 10 degrees for participant 2. As noted in Section 4.3.1 of Data S4, this level of sharing in the genetic pedigree analysis is consistent with a close ancestral relationship. However, due to the randomness with which DNA is inherited, we could not rule out more distant ancestral relationships nor could we rule out the possibility that Burial 56 was a close collateral relative of one or both of the study participants. See Data S4 for a discussion on inferring historical relationships based on IBD sharing.

We therefore searched for historical individuals who appeared in both participant's genealogical family trees in order to identify potential candidates who could be Burial 56. We highlight three historical couples who appear on both trees, meaning that they, and all of their ancestors, are direct ancestors of both participants:

- **Thomas Mattingly** (1623–1664; FamilySearch ID GKC8-RNL) and **Elizabeth McWilliams** (1626–1714; FamilySearch ID GGX6-VZB)
- **John Shercliffe Sr.** (1618–1663; FamilySearch ID LZ99-QGX) and **Anne Spinke** (1618–1678; LBJV-87X)
- **Francis Greene Jr.** (1694–<1761; FamilySearch ID G3LJ-X3W) and **Elizabeth Wheeler** (1693–1758; FamilySearch ID CS8H-VH7)

We systematically considered these individuals and their closest relatives to see if any fit the biological profile of Burial 56, a male, aged approximately 40-49 years old at death (not excluding those who fell slightly outside of this range either way), who died circa 1650-1705, and who could plausibly have been buried in the Chapel Field Cemetery. Additional defining criteria for this individual were his stable isotope bone and tooth collagen values that are consistent with an early childhood migration to, or birth within, the colony. All of the candidates we considered are listed in Data S7N.

During this investigation, we could not identify any candidates in the genealogical trees of Thomas Mattingly, Elizabeth McWilliams, John Shercliffe Sr. or Anne Spinke. The reported parents and siblings of each of these individuals were all born in England, and while a few of their siblings also immigrated to St. Mary's, none had children or grandchildren who fit the profile of Burial 56, according to the data available to us in the FamilySearch database.

The lineage of Francis Greene Jr. and Elizabeth Wheeler was more complicated to investigate, as both individuals had ancestors who immigrated to Maryland several generations in the past, meaning that there were more of their directly ancestral and collateral relatives present in the colony during the period of interest.

We explored all of the lineages with available information to see if any could be considered candidates to fit the profile of Burial 56 (Table S6.1). We identified three individuals who fit the profile for Burial 56, and two additional individuals who were partial matches, but for whom there was missing data, so they could not be ruled out based on their profiles alone. Of these individuals, all but one could be ruled out when we considered the biological profiles the two other members of genetic Family C, with whom Burial 56 shared a first degree relationship:

- **V07C:** A genetically male individual who died around the age of 40-54 years old. Isotopes indicate that he was born in England, and immigrated to Maryland as an adult. He died prior to the construction of the Brick Chapel, and his remains were redeposited in an ossuary when the chapel was built in 1667, providing an indicator of the latest year in which he could have died. Genetic analysis indicates that he shared a first-degree, father-son relationship with Burial 56. Although genetics alone cannot tell us whether he was the father or son of Burial 56, the fact that he was born in England and emigrated to the colonies as an adult–while Burial 56 was born or raised from a young age in Maryland–makes it much more likely that VO7C was the father of Burial 56.
- **Burial 63:**A genetically female individual who died around the age of 25-34 years. She died prior to the construction of the Brick Chapel, between 1634-1667. Isotopes indicate that she was born in England and immigrated to Maryland as an adult. She shared a first-degree relationship with Burial 56. *Note - We did not detect a genetic relationship between Burial 63 or V07C, however due to the limited amount of DNA that could be recovered from these individuals, we cannot rule out that they were related. Therefore when searching for candidates to match the profile of Burial 63, we considered the mother, female siblings, and daughters of candidates who matched the profile of Burial 56.*

A complete list of the individuals who we considered in our search is available in Table S6.1, and the five individuals who either fully or partially matched the profile of Burial 56 are listed below, along with descriptions as to why we consider them to be strong candidates or ultimately ruled them out.

Although our search only revealed a single plausible set of identities that matched the profiles of Burial 56, Burial 63 and V07C, we caution that this identification is only tentative. Our search was limited to data that was available through the FamilySearch database, which, like all historical records, has gaps. Additionally, as this is a publicly curated resource, it is subject to errors. We provided FamilySearch IDs for each of the candidates that we considered in order to document the status of the database at the time of our analysis, and to aid in future studies. Unless otherwise indicated, details about the individuals discussed below (particularly for individuals not in the Greene family) were drawn from the FamilySearch database, and may therefore be subject to error.

Full matches:

- **Leonard Greene** (born c.1636–1638, died: late 1687; FamilySearch ID LC61-QMJ)
    - **Relationship to focal historical couple:** Half-brother of Francis Greene Jr.'s father, Francis Greene Sr.
    - **Degree of separation from historical couple:** 3
    - **Status: Strong candidate.** Leonard Greene was born around 1636–1638 and died in late 1687, around the age of 49-51 years old. He was born and died in Maryland, likely at Greene's Rest plantation [S22]. His godfather was the colony's first governor, Leonard Calvert.

      The Greene family held multiple properties near St. Mary's City. In 1639, Leonard's father patented a 55-acre tract in the Townland of St. Mary's City called Greene's Freehold, where the family lived. They also held Greene's Rest, a plantation located in St. George's Hundred across the St. Mary's River, in addition to a total of 2,400 acres in Charles County known as Greene's Inheritance [S22].

      Leonard's father, Governor Thomas Greene (1609-1651; FamilySearch ID LBLF-6QM), was born in Bobbing, Kent, England and immigrated to St. Mary's aboard The Ark and The Dove in 1634 [S22]. He was appointed the second governor of Maryland in 1647, and died in 1651 at the age of approximately 42 years old, matching the biological profile of V07C. Based on his prominent position in the early governing of St. Mary's City, it is likely that he was buried in the

chapel cemetery, following the destruction of the original chapel in 1645 but prior to the construction of the Brick Chapel. Providing further evidence of this likely burial place is that in his will, Gov. Thomas Greene left 1000 lbs. of tobacco to the Jesuit Priest Thomas Copley and specifically declared himself “a Faithful Christian” [S23]. Both suggest a desire for a chapel field burial although that is not specified in the will.

Gov. Thomas Greene had at least two wives. His first marriage was to Anne Cox (1611-1638), who also traveled aboard The Ark and The Dove [S22]. Theirs is thought to be one of the first Christian marriages performed in the colony of Maryland. Anne was of gentle birth and traveled to the colonies alone, perhaps with a female servant. The couple had two sons, Thomas and Leonard. Thomas (1635–c.1664) died around the age of 30 years old, making him too young to fit the profile for Burial 56.

Anne died around 1638, at the age of approximately 27 years old, possibly during the birth of Leonard, whose exact birth year is unknown (but likely around 1636 to 1638). It is probable that Anne would have been buried at the early Chapel in St. Mary’s. She therefore fits the profile of Burial 63.

Following Anne’s death, it appears that Gov. Thomas Greene sent both of his sons to live in England for some time, as he made a claim in 1644 for land rights for both sons when importing them into the colony [S24 (p. 214-215)].

Gov. Thomas Greene later married Winefred Seyborne (1622–1656), with whom he fathered two sons: Robert Greene (1647–1716) and Francis Greene Sr. (1648–1708). Both died too recently to be candidates for Burial 56 [S22]. The exact date of Thomas and Winefred’s marriage is uncertain; it may have occurred in 1644, the same year his sons Thomas and Leonard returned to the colony. Following Gov. Thomas Greene’s death in 1651, Winefred remarried and moved away, likely leaving Leonard at the family home.

It is also possible Gov. Thomas Greene was briefly married to Millicent Browne in 1643, as he requested a marriage license [S22], though if the marriage occurred, it was short-lived.

- **Ignatius Causine** (1643-1695; FamilySearch ID LTWX-G4B):

    - **Relationship to focal historical couple:** Half-brother of Elizabeth Wheeler's grandmother Mary Ann Edeline Cockshott Causine.
    - **Degree of separation from historical couple:** 4
    - **Status: Ruled out;** Although Ignatius does fit the biological profile of Burial 56, he does not appear to have a first-degree, female relative who fits the biological profile of Burial 63–a female, who died around the age of 25-34 years old, in the pre-Brick Chapel phase (1634-1667). His mother, Jane Dorcas Hicks (1612-1662; FamilySearch ID LZ2G-V56) was approximately 50 years old at the time of her death. Both of his half sisters died after the construction of the Brick Chapel, in 1672 and 1699.
- **Thomas Warren** (1656-1698; FamilySearch ID
    - **Relationship to focal historical couple:** Second cousin of Elizabeth Wheeler
    - **Degree of separation from historical couple:** 5
    - **Status: Ruled out;** Thomas Warren was born in St. Mary's, Maryland in 1656 and died there in 1698. Although his mother, Mary Cole (1654-1683), could fit the profile of Burial 63, he was ruled out as his father, Ignatius Warren (1647-1697) was born in Maryland, and therefore does not fit the isotopic profile of V07C.

Partial Matches (with missing data):

- **John Wheeler** (1654-after 1675; FamilySearch ID LH8J-VY9):
    - **Relationship to focal historical couple:** Uncle of Elizabeth Wheeler
    - **Degree of separation from historical couple:** 2
    - **Status: Ruled out;** John Wheeler was born in Charles, Maryland in 1654 and died some time before 1675. If he died prior to 1704, when the Brick Chapel's cemetery was still in use, he would have been the correct age to fit the profile for Burial 56. However, he was ultimately ruled out as he does not have a first degree female relative who fits the profile for Burial 63. His mother, Mary Ann Edeline Cockshott Causine (1639-1694), was 55 at the time of her death, and his two sisters, Mary (1658-1708) and Winifred (1663-1730) were too old at the time of their

deaths, and died after the Chapel Field cemetery was no longer in use. His father, John Wheeler (born 1930), died some time between 1962 and 1694, making him too old to fit the profile for V07C.

- **Edward Cole** (1657 - before 1717; FamilySearch ID LHB2-N43)
  - **Relationship to focal historical couple:** First cousin, once removed Elizabeth Wheeler
  - **Degree of separation from historical couple:** 4
  - **Status: Ruled out;** Edward Cole was born in St. Mary's, Maryland in 1657. He died some time before 1717. His last child was born in 1696, providing the earliest time of death. He was ruled out, as his father, Robert Cole (1628-1663), died at the age of 35 in England, and therefore does not fit the profile of V07C. Additionally, the Coles were associated with the Catholic Church at Newtown, Maryland, where Edward and other members of the Cole family who immigrated to Maryland were likely buried [S25].
