# Supplementary material for: The genetic legacy of the 17th-century colonial capital of St. Mary’s City
Source: Curr Biol. Author manuscript; Available in PMC 2026 Aug 19. (PMC13487767; doi:10.1016/j.cub.2026.04.046)
Supplement: Supplementary file 7 [file NIHMS2198057-supplement-8.pdf]

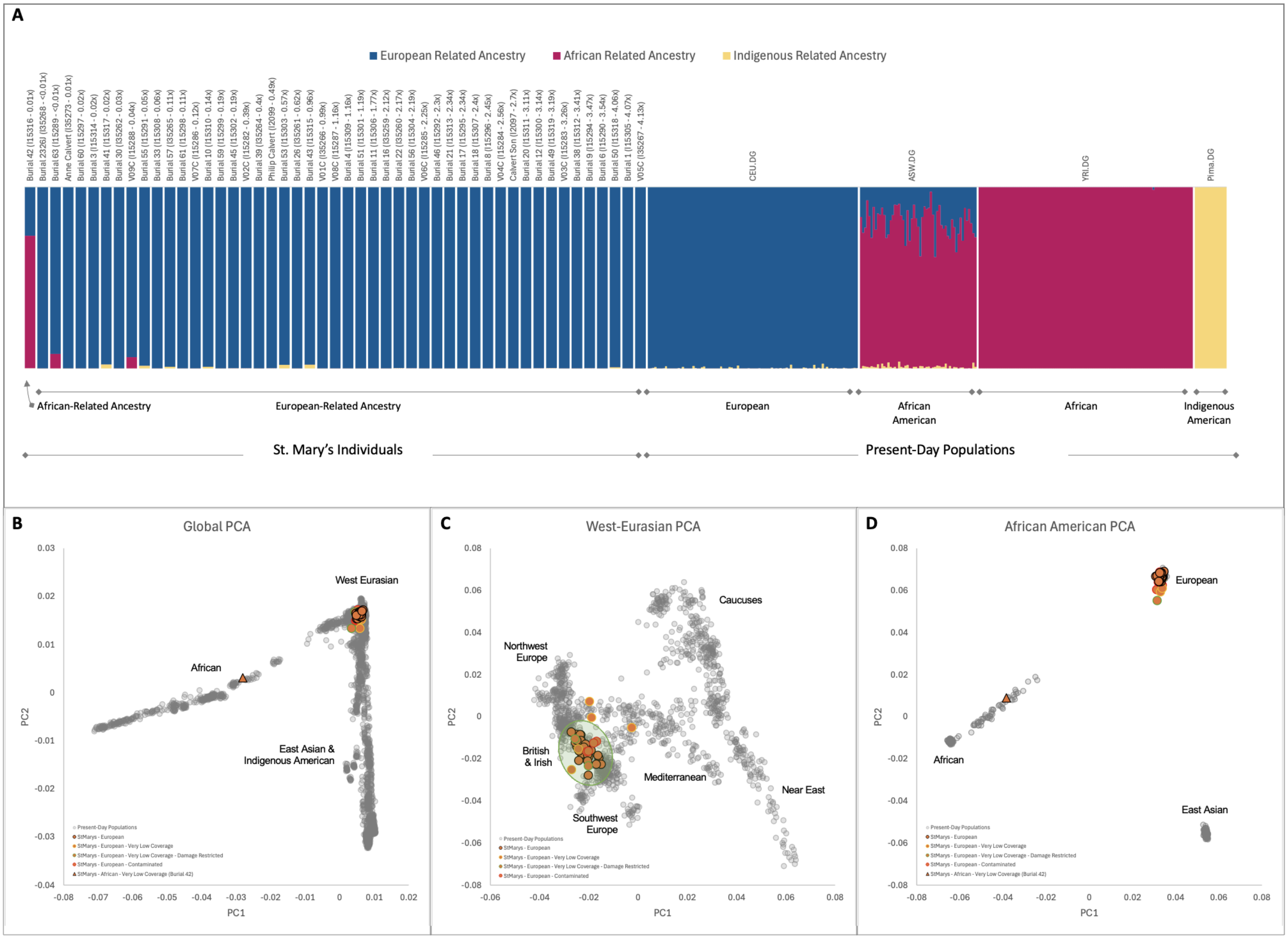

Figure S1. St. Mary's Individuals Ancestry Analysis. Related to STAR Methods. (A) ADMIXTURE analysis of the St. Mary's individuals with present-day individuals with European, African American, African and Indigenous American ancestry (k=3). The St. Mary's individuals are ordered from lowest to highest coverage, except individual I15316, who has substantial African ancestry and is shown on the far left. (B) Global PCA. The St. Mary's individuals projected onto a PCA generated with present-day individuals from across the globe. The approximate location of present-day populations with West Eurasian, African and East Asian and Indigenous American ancestry are indicated. (C) West-Eurasian PCA. The St. Mary's individuals projected onto a PCA generated with present-day individuals from Europe and the Near East. The green circle indicates the approximate location where present-day individuals from Great Britain and Ireland plot on the PCA. (D) African American PCA. The St. Mary's individuals projected onto a PCA generated with present-day individuals from representative populations with European (CEU.DG), African (YRI.DG), African American (ASW.DG) and East Asian (CHB.DG; not shown) ancestry.

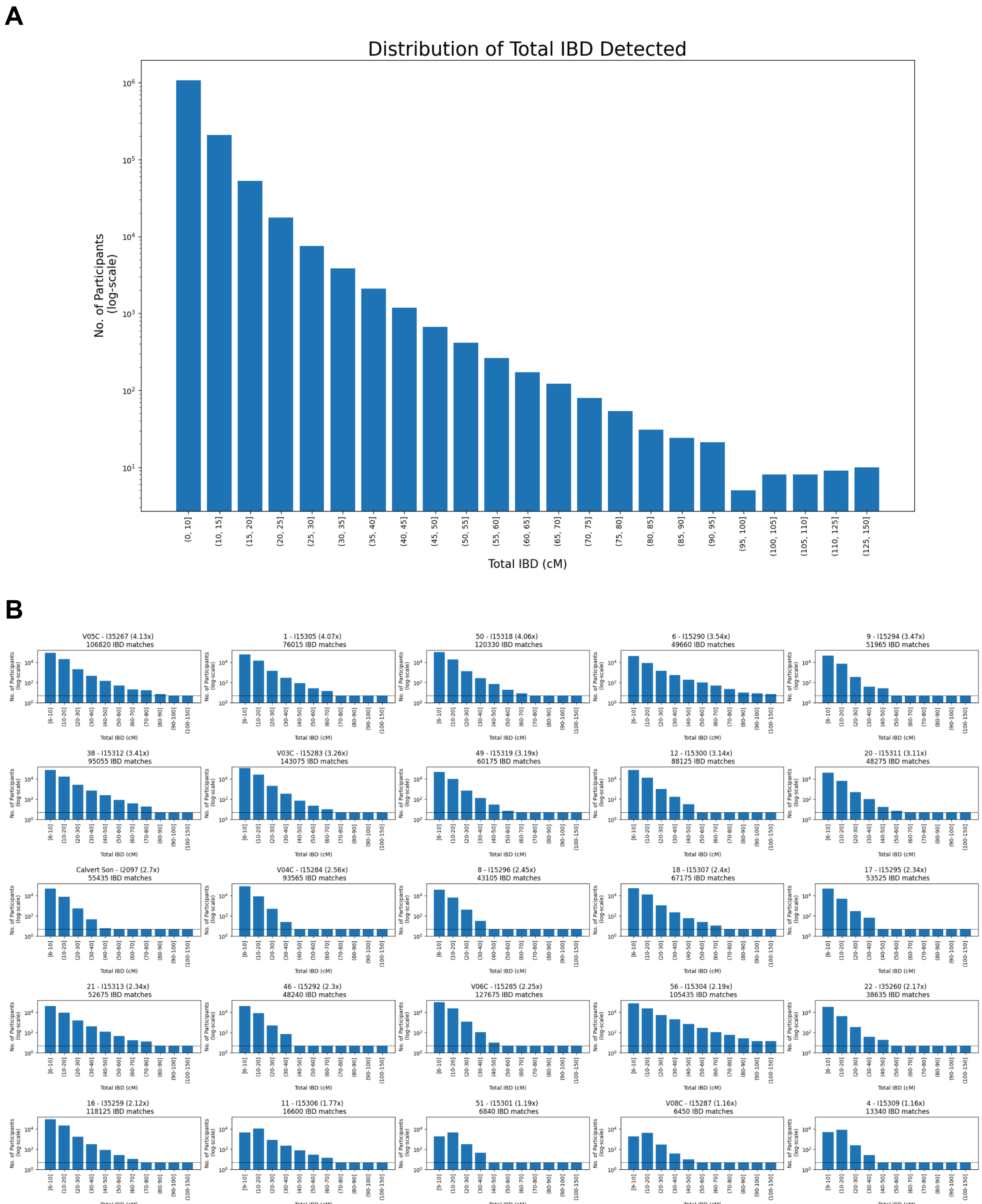

Figure S2. Total IBD sharing with St. Mary's individuals. Related to STAR Methods. (A) Histograms show the number of research participants that share a given amount of total IBD with any St. Mary's individual. In cases where research participants share IBD with multiple St. Mary's individuals, the largest amount of total IBD is reported. Bin sizes were selected to include a minimum of 5 research participants. (B) Histograms show the number of research participants that share a given amount of total IBD with each St. Mary's individual. To maintain research participant anonymity, bins with five or fewer associated research participants are reported as 5, indicated by the black, dotted horizontal line. The minimum IBD segment size considered for historical individuals with coverage of greater than or equal to 2x is 6 cM and 9 cM for historical individuals with coverage less than 2x. The number of IBD matches reported in each figure title is rounded to the nearest 5.

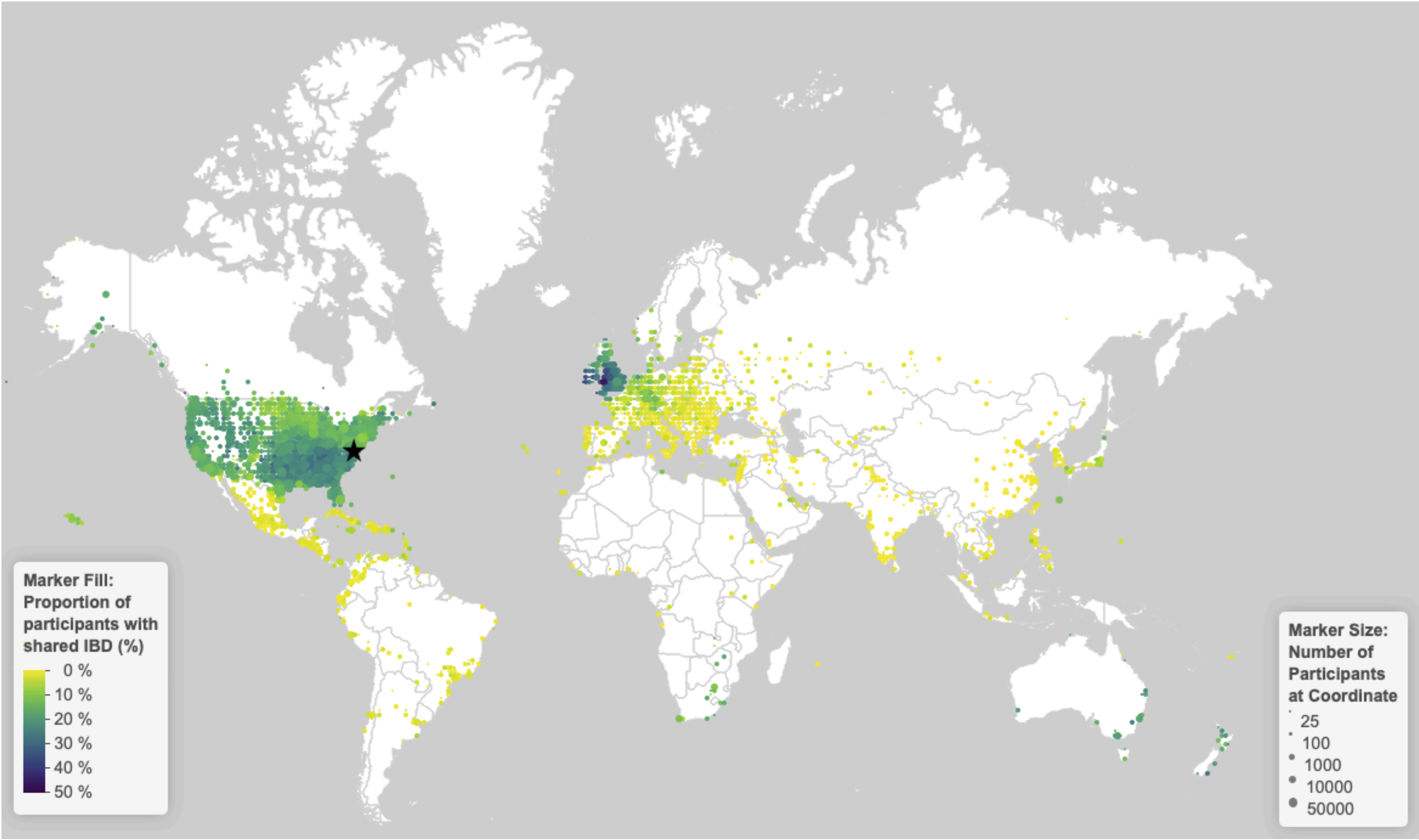

Figure S3. Global Geographic distribution of IBD sharing with St. Mary's individuals. Related to STAR Methods. Marker color indicates the proportion of research participants at each geographic coordinate who share IBD with one or more of the St. Mary's individuals. The size of each marker represents the number of participants at the given geographic coordinate (rounded to the nearest integer). To protect participant privacy, we randomly downsampled to include only 80% of participants and only showed results for coordinates with at least 25 associated participants. The star indicates the location of St. Mary's City, Maryland.

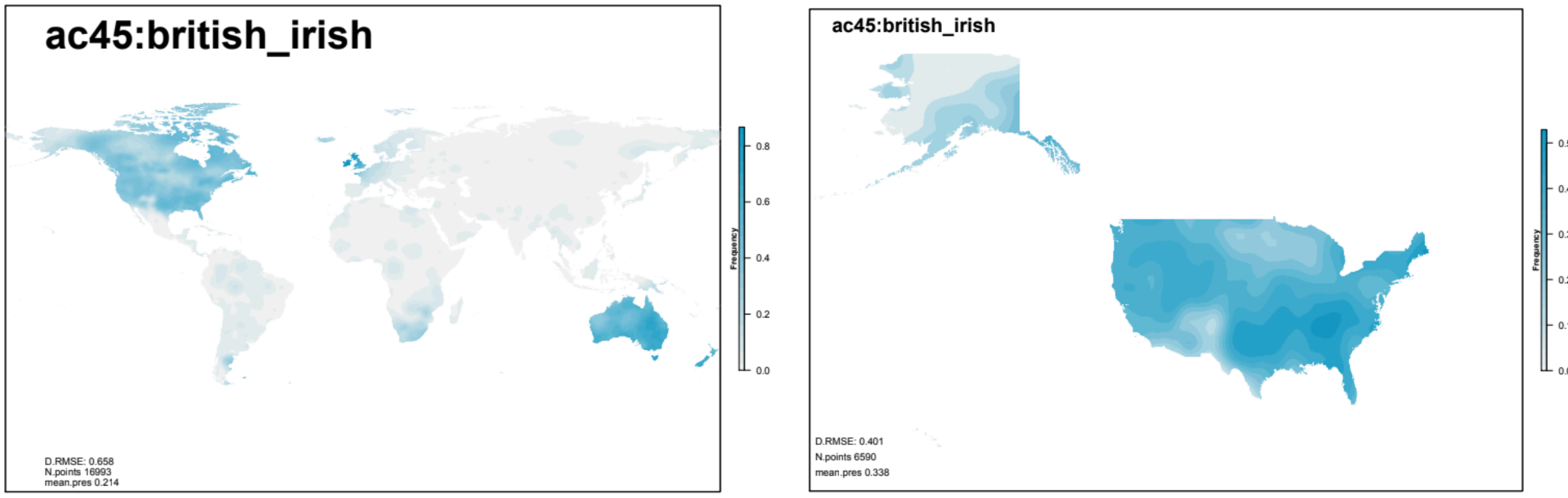

Figure S4. Geographic distribution of 23andMe research participants with British & Irish ancestry. Related to Figure 3. Maps show the predicted British & Irish ancestry proportions (left) globally and (right) in the US using the grandparent birth location of 23andMe research participants. We used Kriging interpolation of the mean proportion of assigned British & Irish ancestry at each geographic coordinate to generate the prediction surfaces.

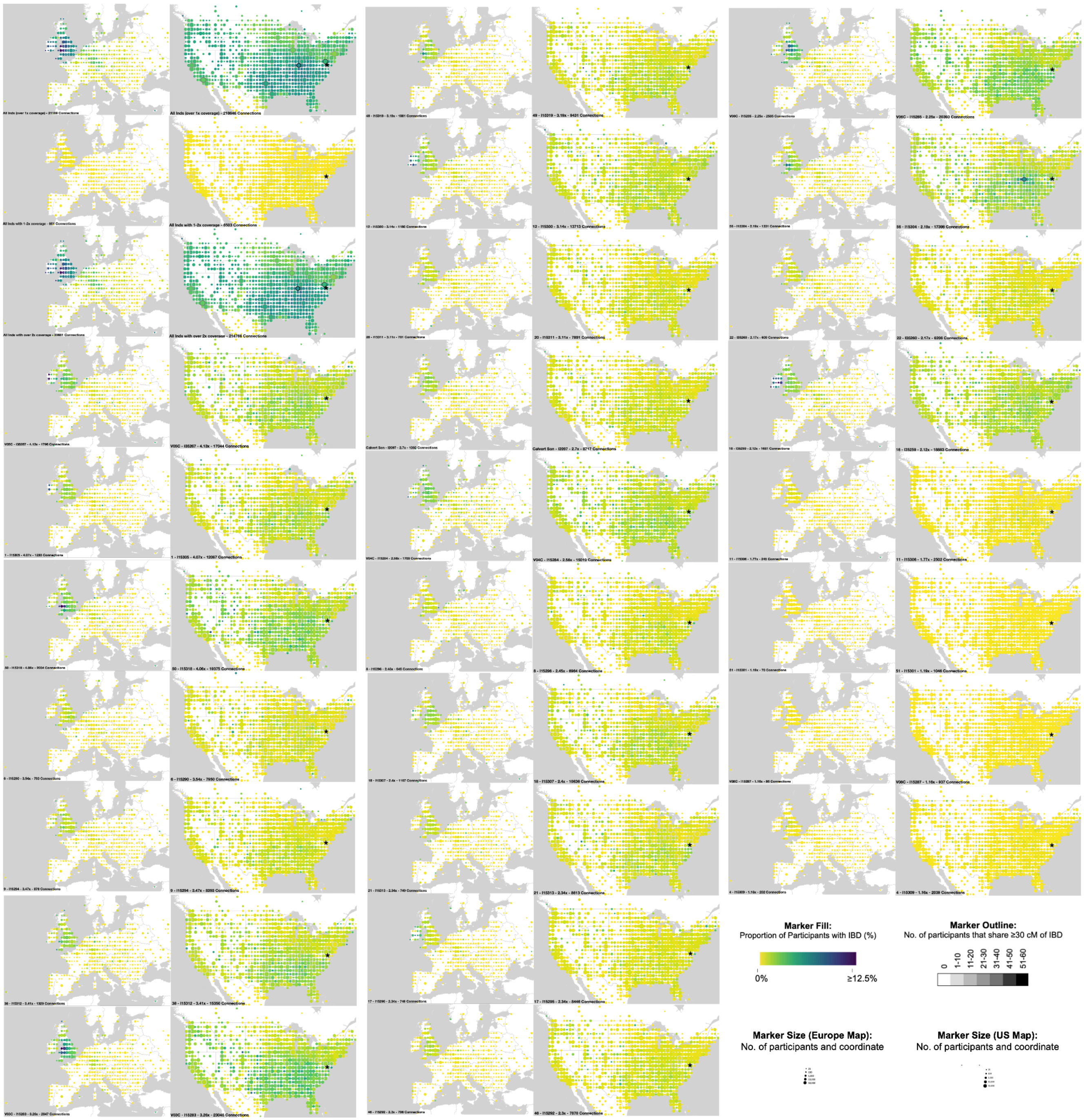

Figure S5. Geographic distribution of IBD sharing with St. Mary's individuals. Related to Figures 3-4. For each St. Mary's individual with over 1x coverage (grouped by coverage and displayed separately), we show the proportion (as indicated by the color of the marker) of research participants at each geographic coordinate who share any amount of IBD. The size of each marker represents the number of participants at the given geographic coordinate (rounded to the nearest integer). To protect participant privacy, we randomly downsampled to include only 80% of participants and only showed results for coordinates with at least 25 associated participants. The total number of participants who share IBD with the St. Mary's individual who are associated with geographic coordinates included in the image is indicated in the figure label, along with the individual ID and their average chromosomal coverage. For each St. Mary's individual we report IBD sharing with (left) research participants with at least 99% European ancestry throughout western and central Europe and (right) research participants in the US. The color of the marker outlines indicates the number of participants at each location who share at least 30 cM of IBD with the St. Mary's individual. The figure title (bottom left of each image) indicates how many research participants shared IBD with (at least one of) the St. Mary's individuals shown.

Supplemental references

S1. Hurry, S.D. (2001). --once the Metropolis of Maryland": The History and Archaeology of Maryland's First Capital (Historic St. Mary's City Commission).

S2. Horn, J. (1979). Servant Immigration to the Chesapeake in the Seventeenth Century. In The Chesapeake in the Seventeenth Century: Essays on Anglo-American Society and Politics. Edited by Thad W. Tate and David L. Ammerman. (University of North Carolina Press), pp. 51–95.

S3. Horn, J. (1994). Adapting to a New World English Society in the Seventeenth-Century Chesapeake (University of North Carolina Press).

S4. Menard, R. (1988). British Migration to the Chesapeake Colonies in the Seventeenth Century. In Colonial Chesapeake Society, Edited by Lois Green Carr, Philip D. Morgan and Jean B. Russo. (University of North Carolina Press), pp. 99–132.

S5. Hall, C.C. (1910). A Relation of Maryland. In Narratives of Early Maryland (Barnes and Noble), pp. 70–77.

S6. Carr, L.G., Menard, R., and Peddicord, L. (1984). Maryland At the Beginning (Maryland State Archives).

S7. Miller, H.M., and Hurry, S.D. (2021). The Archaeology of the Maryland Indians at St. Mary's City and the Interaction of Cultures. In Unearthing St. Mary's City Fifty Years of Archaeology at Maryland's First Capital (University Press of Florida), pp. 95–112.

S8. Seib, R., and Rountree, H.C. (2014). Indians of Southern Maryland (Maryland Historical Society).

S9. Bruwelheide, K.S., Miller, H.M., Owsley, D.W., and Riordan, T.B. (2021). The Lead Coffins of St. Mary's: Burials of the Elite in the Early Chesapeake. In Unearthing St. Mary's City: Fifty Years of Archaeology at Maryland's First Capital (University Press of Florida).

S10. Riordan, T.B., Hurry, S.D., and Miller, H.M. (1995). "A Good Brick Chappell": The Archaeology of the c.1667 Catholic Chapel at St. Mary's City. (Department of Research, Historic St. Mary's City).

S11. Riordan, T.B. (2000). Dig a Grave Both Wide and Deep: An Archaeological Investigation of Mortuary Practices in the 17th-Century Cemetery at St. Mary's City, Maryland (Historic St. Mary's City).

S12. Newman, H.W. (1984). The Flowering of the Maryland Palatinate: An Intimate and Objective History of the Province of Maryland to the Overthrow of Proprietary Rule in 1654, with Accounts of Lord Baltimore's Settlement at Avalon (Genealogical Publishing Com).

S13. Harney, É., Micheletti, S., Bruwelheide, K.S., Freyman, W.A., Bryc, K., Akbari, A., Jewett, E., Comer, E., Gates, H.L., Heywood, L., et al. (2023). The genetic legacy of African Americans from Catoctin Furnace. Science *381*, eade4995. https://doi.org/10.1126/science.ade4995.

S14. Micheletti, S.J., Bryc, K., Ancona Esselmann, S.G., Wilton, P.R., and Freyman, W.A. (2025). Bayesian inference of population structure using identity-by-descent-based stochastic block models. Preprint, https://doi.org/https://doi.org/10.1101/2025.11.26.690810 https://doi.org/https://doi.org/10.1101/2025.11.26.690810.

S15. Huff, C.D., Witherspoon, D.J., Simonson, T.S., Xing, J., Watkins, W.S., Zhang, Y., Tuohy, T.M., Neklason, D.W., Burt, R.W., Guthery, S.L., et al. (2011). Maximum-likelihood estimation of recent shared ancestry (ERSA). Genome Res. *21*, 768–774. https://doi.org/10.1101/gr.115972.110.

S16. Jewett, E.M., and Team, the 23andMe R. (2024). Correcting model misspecification in relationship estimates. Preprint at bioRxiv, https://doi.org/10.1101/2024.05.13.594005 https://doi.org/10.1101/2024.05.13.594005.

S17. Thomas, A., Skolnick, M.H., and Lewis, C.M. (1994). Genomic mismatch scanning in pedigrees. Mathematical Medicine and Biology: A Journal of the IMA *11*, 1–16. https://doi.org/10.1093/imammb/11.1.1.

S18. Jewett, E.M., McManus, K.F., Freyman, W.A., and Auton, A. (2021). Bonsai: An efficient method for inferring large human pedigrees from genotype data. The American Journal of Human Genetics *108*, 2052–2070. https://doi.org/10.1016/j.ajhg.2021.09.013.

S19. Henn, B.M., Hon, L., Macpherson, J.M., Eriksson, N., Saxonov, S., Pe'er, I., and Mountain, J.L. (2012). Cryptic Distant Relatives Are Common in Both Isolated and Cosmopolitan Genetic Samples. PLOS ONE *7*, e34267. https://doi.org/10.1371/journal.pone.0034267.

S20. Seidman, D.N., Shenoy, S.A., Kim, M., Babu, R., Woods, I.G., Dyer, T.D., Lehman, D.M., Curran, J.E., Duggirala, R., Blangero, J., et al. (2020). Rapid, Phase-free Detection of Long Identity-by-Descent Segments Enables Effective Relationship Classification. The American Journal of Human Genetics *106*, 453–466. https://doi.org/10.1016/j.ajhg.2020.02.012.

S21. Freyman, W.A., McManus, K.F., Shringarpure, S.S., Jewett, E.M., Bryc, K., the 23andMe Research Team, and Auton, A. (2020). Fast and robust identity-by-descent inference with the templated positional Burrows-Wheeler transform. Molecular Biology and Evolution. https://doi.org/10.1093/molbev/msaa328.

S22. Dr. Lois Green Carr, Men's Career Files MSA SC 5094 Image No: sc5094-1628-5 https://msa.maryland.gov/megafile/msa/speccol/sc5000/sc5094/001000/001628/html/sc5094-1628-5.html.

S23. Archives of Maryland, Volume 0010, Page 0088 - Judicial and Testamentary Business of the Provincial Court, 1649/50-1657 https://msa.maryland.gov/megafile/msa/speccol/sc2900/sc2908/000001/000010/html/am10--88.html.

S24. Newman, H.W. (1984). The Flowering of the Maryland Palatinate: An Intimate and Objective History of the Province of Maryland to the Overthrow of Proprietary Rule in 1654, with Accounts of Lord Baltimore's Settlement at Avalon (Genealogical Publishing Com).

S25. Carr, L.G., Menard, R.R., and Walsh, L.S. (2017). Robert Cole's World: Agriculture and Society in Early Maryland (UNC Press Books).
